# Mean-shift exploration in shape assembly of robot swarms

**DOI:** 10.1038/s41467-023-39251-5

**Published:** 2023-06-13

**Authors:** Guibin Sun, Rui Zhou, Zhao Ma, Yongqi Li, Roderich Groß, Zhang Chen, Shiyu Zhao

**Affiliations:** 1 School of Automation Science and Electrical Engineering, Beihang University, Beijing, China; 2 School of Engineering, Westlake University, Hangzhou, China; 3 Department of Automatic Control and Systems Engineering, The University of Sheffield, Sheffield, UK; 4 Department of Automation, Tsinghua University, Beijing, China; 5 Research Center for Industries of the Future, Westlake University, Hangzhou, China; 6 Key Laboratory of Coastal Environment and Resources of Zhejiang Province, Westlake University, Hangzhou, China; 7 Westlake Institute for Advanced Study, Hangzhou, China

**Keywords:** Electrical and electronic engineering, Complex networks

## Abstract

The fascinating collective behaviors of biological systems have inspired extensive studies on shape assembly of robot swarms. Here, we propose a strategy for shape assembly of robot swarms based on the idea of mean-shift exploration: when a robot is surrounded by neighboring robots and unoccupied locations, it would actively give up its current location by exploring the highest density of nearby unoccupied locations in the desired shape. This idea is realized by adapting the mean-shift algorithm, which is an optimization technique widely used in machine learning for locating the maxima of a density function. The proposed strategy empowers robot swarms to assemble highly complex shapes with strong adaptability, as verified by experiments with swarms of 50 ground robots. The comparison between the proposed strategy and the state-of-the-art demonstrates its high efficiency especially for large-scale swarms. The proposed strategy can also be adapted to generate interesting behaviors including shape regeneration, cooperative cargo transportation, and complex environment exploration.

## Abstract (web summary)

Achieving shape assembly behaviour in robot swarms with adaptability and efficiency is challenging. Here, Sun et. al. propose a strategy based on an adapted mean-shift algorithm, thus realizing complex shape assembly tasks such as shape regeneration, cargo transportation, and environment exploration.

## Introduction

In nature, groups of insects and animals can self-assemble various spatial shapes that are functional for the groups adapting to the environments^1–3^. As a remarkable example, army ants can assemble shapes to transport food cooperatively or construct bridges using their bodies to overcome spatial gaps^4,5^. These shape assembly behaviors are generated spontaneously by local interactions among the individuals. They exhibit strong adaptability to individual faults and can be easily scaled up to groups of thousands or millions of individuals^1,3^.

The fascinating collective behaviors of biological systems have inspired extensive studies on shape assembly of robot swarms^6–9^. One class of strategies widely studied in the literature are based on goal assignment in either centralized or distributed ways^10–12^. Once a swarm of robots are assigned unique goal locations in a desired shape, the consequent task is simply to plan collision-free trajectories for the robots to reach their goal locations^10^ or conduct distributed formation control based on locally sensed information^6,13,14^. It is notable that centralized goal assignment is inefficient to support large-scale swarms since the computational complexity increases rapidly as the number of robots increases^15,16^. Moreover, when robots fail to function normally, additional algorithms for fault-tolerant detection and goal re-assignment are required to handle such situations^17^. As a comparison, distributed goal assignment can support large-scale swarms by decomposing the centralized assignment into multiple local ones^11,12^. It also exhibits better robustness to robot faults. However, since distributed goal assignments are based on locally sensed information, conflicts among local assignments are inevitable and must be resolved by sophisticated algorithms such as local task swapping^11,12^.

Another class of strategies for shape assembly that have also attracted extensive research attention are free of goal assignment^18–21^. For instance, the method proposed in ref. ^18^ can assemble complex shapes using thousands of homogeneous robots. An interesting feature of this method is that it does not rely on external global positioning systems. Instead, it establishes a local positioning system based on a small number of pre-localized seed robots. As a consequence of the local positioning system, the proposed edge-following control method requires that only the robots on the edge of a swarm can move while those inside must stay stationary. The method in ref. ^19^ can generate swarm shapes spontaneously from a reaction-diffusion network similar to embryogenesis in nature. However, this method is not able to generate user-specified shapes precisely. The method in ref. ^21^ can aggregate robots on the frontier of shapes based on saliency detection. The user-defined shape is specified by a digital light projector. An interesting feature of this method is that it does not require centralized edge detectors. Instead, edge detection is realized in a distributed manner by fusing the beliefs of a robot with its neighbors. However, since the robots cannot self-localize themselves relative to the desired shape, they make use of random walks to search for the edges, which would lead to random trajectories. Another class of methods that do not require goal assignment is based on artificial potential fields^22–25^. One limitation of this class of methods is that robots may easily get trapped in local minima, making it difficult to assemble nonconvex complex shapes.

Here, we propose a strategy for shape assembly of robot swarms based on the idea of mean-shift exploration: when a robot is surrounded by neighboring robots and unoccupied locations, it would actively give up its current location by exploring the highest density of nearby unoccupied locations in the desired shape. This idea does not rely on goal assignment. It is realized by adapting the mean-shift algorithm^26–28^, which is an optimization technique widely used in machine learning for locating the maxima of a density function. Moreover, a distributed negotiation mechanism is designed to allow robots to negotiate the final desired shape with their neighbors in a distributed manner. This negotiation mechanism enables the swarm to maneuver while maintaining a desired shape based on a small number of informed robots. The proposed strategy empowers robot swarms to assemble nonconvex complex shapes with strong adaptability and high efficiency, as verified by numerical simulation results and real-world experiments with swarms of 50 ground robots. The strategy can be adapted to generate interesting behaviors including shape regeneration, cooperative cargo transportation, and complex environment exploration.

## Results

The proposed shape assembly strategy consists of three components. The first is a human-swarm graphical interface that can specify user-defined shapes. The second is a distributed negotiation process that can autonomously reach an agreement among the robots on the final location and orientation of the swarm shape. The third, which is the core of the proposed strategy, is a distributed control algorithm. The three components are detailed as follows.

### A human-swarm graphical interface

**Fig. 1:**
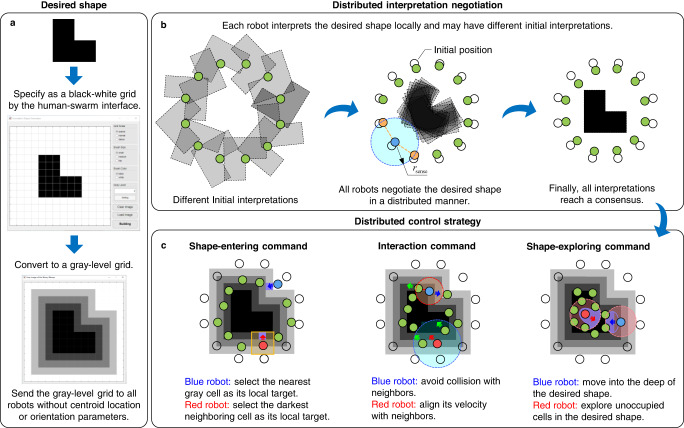
An illustration of the proposed shape assembly strategy. **a** The human-swarm interface. The desired shape is specified via the interface and sent to all the robots as a nonparametric grid. **b** The robots have different initial interpretations of the translation and orientation of the desired shape. They gradually reach a consensus via the proposed distributed negotiation algorithm. **c** Examples to illustrate the three control commands in the proposed control strategy. Details of the algorithms can be found in Methods and Sections 1 to 3 in the Supplementary Information.

The first step of shape assembly is to specify a desired geometric shape. We designed a human-swarm interface that allows a human operator to specify the desired shape by drawing or loading a binary graphical image (Fig. 1a). The graphical image is converted to a black-white binary grid, where the black cells in the grid correspond to the locations that should be occupied by the robots. The binary grid is then converted to a grayscale grid by the distance transformation algorithm^29^ so that the influence of the desired shape is gradually expanded and the robots can move into the shape more smoothly (Fig. 1a). With this human-swarm interface, the operator can specify the desired shape in a graphical way without specifying the physical parameters of the grid. The parameters such as the physical size of each cell can be autonomously generated. Moreover, the number of cells in the grid does not have to be the same as the number of robots because the proposed method can handle mismatches between the grid and robot numbers (Fig. 4 as shown later). Although the desired shape is represented as a discrete grid, the robots can move freely across the boundaries of the cells in the grid. More information can be found in Methods and Section 1 in the Supplementary Information.

### Distributed negotiation on the desired shape

Once the grayscale grid has been generated by the human-swarm interface, it is sent to all the robots and stored in each robot’s memory. The memory sizes of the grids used in our work are around tens of kilobytes (details are given in Section 1 in the Supplementary Information), which is affordable for mainstream embedded computers nowadays. The Euclidean parameters of the desired shape including the position or orientation are not assigned manually. Instead, the robots negotiate with each other to autonomously determine them in a distributed manner. In particular, each robot first interprets the desired shape based on its local interest. For example, every robot may initially interpret itself as the center of the desired shape (Fig. 1b), which is locally optimal for each robot in the sense that they do not need to move because they are already inside the desired shape. While the initial interpretations of different robots may conflict, each robot negotiates its interpretation with its neighbors through local wireless communication by a distributed consensus algorithm. With the negotiation algorithm, all the robots would eventually reach a consensus on the translation and orientation of the final desired shape (Fig. 1b). By introducing a small number of informed robots, the swarm can reach a consensus on a user-specified trajectory of the desired shape. More information about shape negotiation is given in Methods and Section 2 and Section 6.1 in the Supplementary Information.

### Distributed control of shape assembly

The aim of each robot is to move into the desired shape. This process is highly dynamic because its goal location is time-varying due to the dynamical convergence process of its interpretation and, more importantly, the avoidance of inter-robot collision or goal location competition. In our method, each robot executes the same control strategy based on its locally sensed information so as to enter the desired shape while avoiding collision. In particular, the velocity command for the *i*th robot is

$${{{{{{{{\bf{v}}}}}}}}}_{i}={{{{{{{{\bf{v}}}}}}}}}_{i}^{{{{{{{{\rm{ent}}}}}}}}}+{{{{{{{{\bf{v}}}}}}}}}_{i}^{\exp }+{{{{{{{{\bf{v}}}}}}}}}_{i}^{{{{{{{{\rm{int}}}}}}}}},$$

where ${{{{{{{{\bf{v}}}}}}}}}_{i}^{{{{{{{{\rm{ent}}}}}}}}},\,{{{{{{{{\bf{v}}}}}}}}}_{i}^{\exp }$, and ${{{{{{{{\bf{v}}}}}}}}}_{i}^{{{{{{{{\rm{int}}}}}}}}}$ represent the shape-entering, shape-exploring, and interaction velocity commands, respectively. The roles of the three velocity commands are explained as follows, whereas their mathematical expressions are given in Methods.

The shape-entering command ${{{{{{{{\bf{v}}}}}}}}}_{i}^{{{{{{{{\rm{ent}}}}}}}}}$ aims to steer robot *i* to the desired shape by seeking the darkest grid cells around them (Fig. 1c and Methods). More specifically, when robot *i* is too far away from the desired shape, ${{{{{{{{\bf{v}}}}}}}}}_{i}^{{{{{{{{\rm{ent}}}}}}}}}$ would drive the robot toward the closest gray cell. When robot *i* has entered the area of the gray cells, ${{{{{{{{\bf{v}}}}}}}}}_{i}^{{{{{{{{\rm{ent}}}}}}}}}$ would push the robot toward one of the neighboring gray cells that has the lowest gray level. In this way, the robot can gradually reach a black cell in the desired shape. It is notable that ${{{{{{{{\bf{v}}}}}}}}}_{i}^{{{{{{{{\rm{ent}}}}}}}}}$ would vanish once a robot steps into the desired shape area. In this case, the robot would stop moving and hence block those robots behind. To resolve this problem, the following velocity command ${{{{{{{{\bf{v}}}}}}}}}_{i}^{\exp }$ is designed so that the robots can continue moving into the deep of the desired shape.

The shape-exploring command ${{{{{{{{\bf{v}}}}}}}}}_{i}^{\exp }$, which is realized by an adapted version of the mean-shift algorithm^27^, aims to push each robot into the desired shape and then explore unoccupied cells inside the desired shape (Fig. 1c and Methods). More specifically, there are two cases. In the first case where robot *i* is near the edge of the desired shape so that the cells within its sensing radius are either black or gray, ${{{{{{{{\bf{v}}}}}}}}}_{i}^{\exp }$ would drive the robot toward the highest density of black cells. In this way, the robot is attracted into the shape. In the second case where robot *i* is already inside the desired shape so that the cells within its sensing radius are all black, ${{{{{{{{\bf{v}}}}}}}}}_{i}^{\exp }$ would drive the robot toward the direction with the highest density of the unoccupied black cells. A cell is defined as unoccupied if the distance between its center and any robot is greater than half of the robot’s collision avoidance radius (F 3B). The shape-exploring command is the key to resolving inter-robot competition. When a robot is surrounded by neighboring robots and unoccupied locations, it would actively give up its current location by exploring the highest density of nearby unoccupied locations.

The interaction command ${{{{{{{{\bf{v}}}}}}}}}_{i}^{{{{{{{{\rm{int}}}}}}}}}$ consists of two sub-terms (Fig. 1c and Methods). The first is a collision-avoidance term that generates repulsive velocity commands when robot *i* is too close to its surroundings. The second is a velocity-alignment term that aligns the velocity of robot *i* with its neighbors. The velocity-alignment term plays two roles. First, it can reduce velocity mismatches among the robots to reduce the chances of inter-robot collision. Second, since the desired shape may maneuver across in the motion space, the velocity-alignment term is necessary for robot *i* to track the velocity of the moving desired shape.

### Platforms and experimental setup

**Fig. 2:**
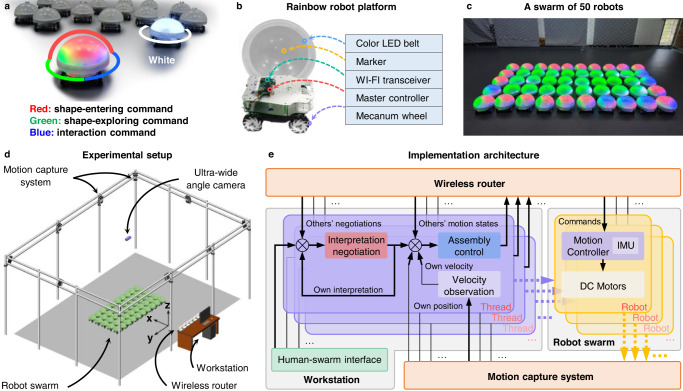
Platforms and implementation setup. **a** The specifically designed Rainbow robots. Each robot can show different colors to reflect the ingredients of its control commands. **b** Hardware components of robot platform. See Section 5.1 in the Supplementary Information for more details. **c** A swarm of 50 Rainbow robots. **d** An illustration of the experimental setup. The detailed functions of each experimental element are given in Section 5.2 in the Supplementary Information. **e** The multi-thread implementation architecture. Each robot corresponds to a unique control thread in the workstation, and the threads are executed in a parallel way.

We implemented the proposed strategy on a swarm of 50 holonomic wheeled robots (Fig. 2a, b). Details of the robotic platforms are given in Section 5.1 in the Supplementary Information. Each robot can exhibit its velocity command by using a LED belt inside the robot’s dome. In particular, each LED can show four colors: red, green, blue, and white (Fig. 2a). The numbers of LEDs showing the four colors correspond to the magnitudes of ${{{{{{{{\bf{v}}}}}}}}}_{i}^{{{{{{{{\rm{ent}}}}}}}}},{{{{{{{{\bf{v}}}}}}}}}_{i}^{\exp },{{{{{{{{\bf{v}}}}}}}}}_{i}^{{{{{{{{\rm{int}}}}}}}}}$, and **v**_*i*_, respectively (Section 5.1 in the Supplementary Information). The experiments were conducted in an indoor environment with the support of a motion capture system (Fig. 2c, d). To implement the proposed strategy, each robot needs to acquire certain information about its neighbors and sense its surroundings to identify unoccupied cells and obstacles. These functions are realized through a parallel multi-thread system in a distributed manner (Fig. 2e). More specifically, each robot is assigned an independent control thread running on the workstation and communicates with its thread using wireless routers. Although all the information is available in the workstation, the control command for each robot merely uses the local information that each robot is supposed to have. Details of the experimental setup are given in Section 5.2 in the Supplementary Information.

### Complex shape assembly

**Fig. 3:**
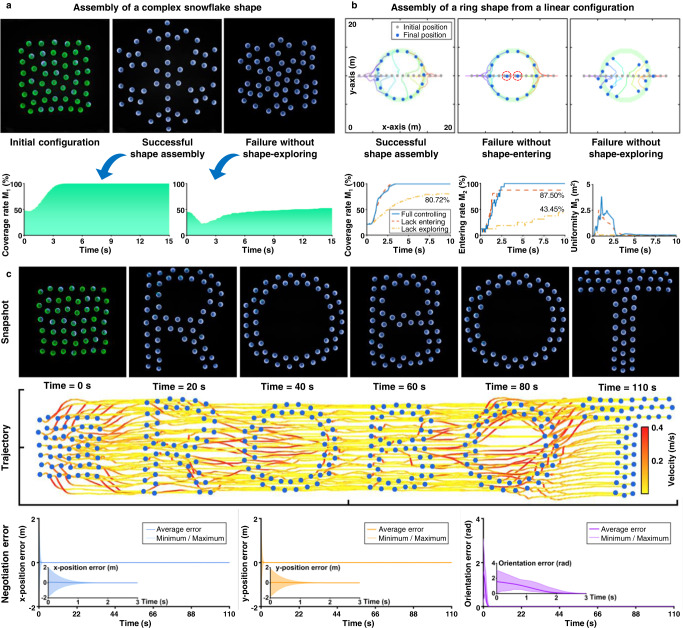
Complex shape assembly by the proposed strategy. **a** Experimental results of 50 real Rainbow robots assembling a complex snowflake shape. The shape-exploring control term is necessary since the shape assembly task would fail in the absence of the term as shown in the rightmost subfigure. **b** Numerical simulation of 16 robots assembling a ring shape starting from an initial linear configuration. Both the shape-exploring and shape-entering control terms are necessary since shape assembly would fail in the absence of either of them. **c** Experimental results of 50 real Rainbow robots assembling different shapes in a sequence. The distributed negotiation process among the robots converges fast. The shape switches smoothly due to the ability of active exploration and independence of goal assignment of the proposed strategy. See Supplementary Movies 1 and 2.

The proposed strategy is able to assemble nonconvex complex shapes. One representative complex shape is the snowflake as shown in Fig. 3a. The snowflake shape has 6 major branches and 18 minor branches, making the shape highly nonconvex and challenging to assemble. The proposed strategy can assemble the snowflake shape, which is quantitatively verified by the fact that the coverage rate converges to 100% (Fig. 3a). The coverage rate and other metrics to evaluate the assembly performance are defined in Methods. The shape-exploring control term of the strategy plays a key role in this process. In particular, when this term is disabled, the swarm fails to assemble the snowflake shape since many robots get trapped in local minima and the coverage rate drops sharply to 75% (Fig. 3a). The reason for the failure is that a robot would stop moving and hover near the boundary once it has entered the desired shape in the absence of this term. As a result, it may block those robots behind from entering the shape (Fig. 3b). By contrast, with this term, a robot would continue moving toward the deep of the desired shape after passing the boundary so that those robots behind it can enter the shape smoothly (Fig. 3b). In addition to the shape-exploring term, the shape-entering term in the proposed strategy also plays a necessary role to steer all the robots into the desired shape. As shown in Fig. 3b, not all the robots can enter the shape in the absence of this shape-entering term even though the shape can still be fulfilled by a subset of the swarm. The proposed strategy exhibits smooth swarming motion in shape forming and switching tasks. As shown in the experimental results in Fig. 3c, the swarm can assemble different nonconvex shapes and switch from one to another smoothly.

**Fig. 4:**
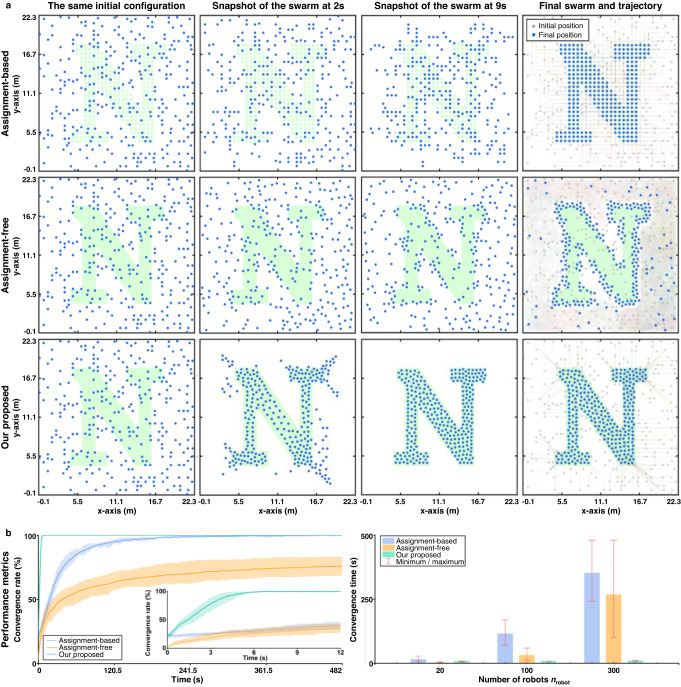
Comparison between the proposed method, the decentralized assignment-based method in ref. ^12^ and the decentralized assignment-free method in ref. ^21^. **a** Snapshots and trajectories of the shape assembly processes by the three methods. There are 300 robots assembling the shape of “N''. **b** Statistics of convergence rate and convergence time. Each average value is calculated based on 10 trials. For the proposed method and the assignment-based one in ref. ^12^, the convergence rate is defined as the ratio between the number of robots that are inside the shape and the total number of robots. The convergence time is defined as the time when the convergence rate is equal to 100%. For the assignment-free method in ref. ^21^, the convergence rate is defined as the ratio between the number of robots that have reached the edge and the total number of robots. The width of the border, a parameter in the method in ref. ^21^, is set to zero so that the robots can aggregate evenly around the shape as much as possible. Since it is difficult for all the robots evenly distribute along the edge, the convergence time is defined as the time when the convergence rate reaches 70%. The parameters of the three algorithms are given in Supplementary Table 2.

In the following, the efficiency of the proposed method is compared with that of two state-of-the-art methods. The first is the assignment-based method in ref. ^12^ and the second is the assignment-free method in ref. ^21^. Both of the methods are decentralized. The method in ref. ^12^ and our proposed method require external global positioning, whereas the one in ref. ^21^ does not. The comparison results are presented in Fig. 4 and Supplementary Fig. 8. It is shown that, given the same initial configurations, the three methods have similar convergence times when the number of robots is as small as 20. However, as the number of robots increases, the convergence times of the state-of-the-art methods increase rapidly while that of the proposed one increases slightly. Specifically, when the number of robots is as large as 300, the convergence time of the proposed method is at least 20 times shorter than the others. The reason why the method in ref. ^12^ requires longer convergence time is that it executes local goal swaps constantly and the robots must move along the grid lines. By contrast, the proposed method does not rely on goal assignment due to the mean-shift exploration control. It also allows the robots to move continuously in the working space. The merit of the method in ref. ^21^ is that it does not rely on any global or local positioning system. However, since the robots do not know their positions relative to the desired shape, they make use of random walks to search for the edges of the desired shape, which would lead to random and long trajectories.

### Adaptability to swarm scale variants

**Fig. 5:**
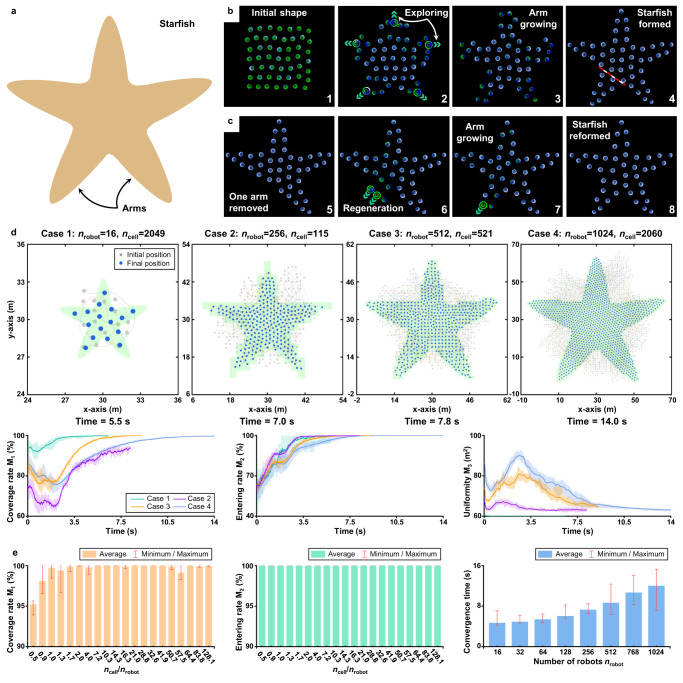
Adaptability of the proposed strategy. **a** The desired shape is a starfish^33^. **b**, **c** Experimental results of 50 real Rainbow robots assembling a starfish shape. If one arm of the starfish shape is removed, the swarm spontaneously grows a new arm without any goal re-assignment or fault-tolerant control thanks to the shape-exploring control term. See Supplementary Movie 3. **d** It is shown by numerical simulation that the strategy shows stable performance in the presence of the mismatch between *n*_robot_ and *n*_cell_, quantitatively verified by the three metrics of coverage rate, entering rate, and uniformity. **e** Statistical results of 512 simulations of the starfish assembly task given mismatched values of *n*_robot_ and *n*_cell_. In each simulation, *n*_robot_ and *n*_cell_ take values respectively from {16, 32, 64, 128, 256, 512, 768, 1024} and {115, 521, 920, 1321, 1623, 2049, 2060, 2682}. Each pair of *n*_robot_ and *n*_cell_ further corresponds to eight trials with random initializations (including robots' locations and their interpretations of the desired shape). The 8 × 8 × 8 = 512 statistical results show that the proposed strategy exhibits stable performance in terms of the evaluation metrics in the presence of strong mismatches between *n*_robot_ and *n*_cell_.

The proposed strategy is adaptive to the variant of robot number in a swarm. When some robots leave the swarm, the remaining robots can autonomously assemble the desired shape without any goal re-assignment or fault-tolerant control. A representative example is the shape regeneration behavior as demonstrated in Fig. 5, where the desired geometric shape is a starfish. After one arm of the starfish shape has been removed, the rest of the swarm spontaneously grows a new arm and assembles the starfish shape again. The adaptability is due to the shape-exploring term, by which the robots can actively search unoccupied cells and hence replace the roles of the removed robots.

Notably, the newly generated starfish has less number of robots than the original one, verifying that the proposed strategy can assemble the same shape with different numbers of robots. In fact, the strategy exhibits stable performance in the presence of mismatches between the number of robots, denoted as *n*_robot_, and the number of black cells in the desired shape, denoted as *n*_cell_ (Fig. 5d). For the starfish example, it is shown by the statistical results in Fig. 5e that the proposed strategy can successfully assemble the desired shape with a wide range of *n*_cell_/*n*_robot_. Specifically, the coverage rate of robots for the desired shape is greater than 93% and the entering rate remains 100% when the ratio *n*_cell_/*n*_robot_ varies from 0.45 to 128.06. As a consequence, the strategy avoids the requirement of the condition *n*_robot_ = *n*_cell_, a condition widely adopted in shape assembly methods^11^, and hence makes the strategy more adaptive. In addition, when the swarm scale increases from *n*_robot_ = 16 to 1024, the convergence time of the entire swarm increases mildly as shown in the rightmost subfigure of Fig. 4E, verifying the high motion efficiency of the strategy.

### Cooperative transportation and shape maneuvering

**Fig. 6:**
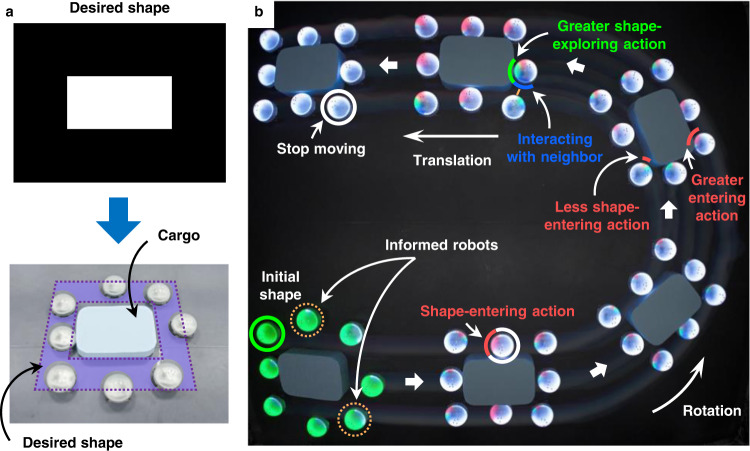
Cooperative cargo transportation by a swarm of eight real Rainbow robots. **a** The desired shape is specified as a hollow rectangle centered at the cargo. **b** The process of cooperative transportation by the swarm. When the robots assemble the desired shape, they would encircle the cargo tightly. Since the desired shape is moving, the robots would transport the cargo while tracking the desired moving shape. The entire process is led by two informed robots. The color of each robot indicates its velocity command. See Supplementary Movies 4.

In nature, ants can surround and transport a piece of food that is much larger than their individual body size in a cooperative manner^4^. The proposed strategy can be applied to cooperative cargo transportation. To do that, we can specify a hollow shape centered at a cargo (Fig. 6a). When the robots assemble the hollow shape, they would encircle the cargo tightly. The consequent task is to steer the swarm to move collectively while maintaining the desired shape. In particular, we introduce a small number of informed robots that know the desired translational and rotational trajectory of the shape. The informed robots have counterparts in ant swarms^4^, where some leader ants can guide the others to transport food to a goal location. During the inter-robot negotiation process, the informed robots play a stubborn role by insisting on their knowledge of the desired trajectory. The interpretations of the rest uninformed robots would gradually converge to the informed ones so that all the robots reach a consensus on the desired trajectory of the shape (see the details of the algorithm in Section 2 in the Supplementary Information). While the robots attempt to assemble the desired moving shape, they automatically transport the cargo encircled in the center (Fig. 6b). The velocity alignment component in the interaction control term of the proposed strategy is essential for all the robots tracking the maneuvering swarm shape.

The number of informed robots is required to be at least one. The more informed robots there are, the faster the negotiation process can converge. As shown in Supplementary Figure 6, seven informed robots are randomly selected out of 128 ones, which can reach a consensus on the time-varying translation and orientation of the desired shape efficiently. As a consequence, the swarm can maneuver while the overall shape varies. Being able to track moving shapes is an important feature of the proposed strategy. By contrast, the state-of-the-art methods for homogeneous swarms are only applicable to static shapes^10,12,18,19,30^. Although there is a rich body of control-theoretic methods that can achieve maneuvering formations^14,31,32^, these methods require goal assignments or unique robot identities.

### Complex environment exploration

**Fig. 7:**
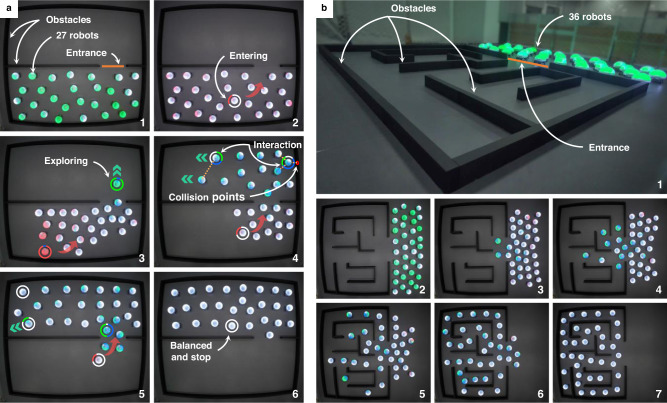
Environment exploration by the proposed strategy. **a** Experiments of 27 real Rainbow robots flooding into a room through a narrow passage. This scenario mimics the process of pedestrians entering a passenger elevator: The pedestrians that enter the elevator first should move into the deep rather than staying near the entrance to block the people behind. **b** Experiments of 36 real Rainbow robots exploring a complex maze without getting deadlocked at any nonconvex corners. See Supplementary Movies 5 and 6.

The proposed shape assembly strategy can be applied to environment exploration tasks, in which a swarm must evenly fulfill an environment while avoiding obstacles. As a representative example, a swarm can flood into a room through a narrow passage without getting trapped at the entrance (Fig. 7a). This example mimics the process of pedestrians entering a passenger elevator, which well demonstrates the idea of mean-shift exploration of the proposed strategy: The pedestrians that enter the elevator first should move into the deep rather than staying near the entrance to block the people behind.

The proposed strategy can also be used to explore more complex environments such as a maze as shown in Fig. 7b. Although there are many nonconvex corners in the maze, the swarm can successfully fulfill the maze without getting deadlocked at any corner, verifying the strong exploration ability of the proposed strategy. The strategy can well balance the exploration speed and inter-robot connectivity. That is, a robot inside the desired shape would stop exploring when it has no neighbors inside its sensing radius. As a result, the robots would not explore too fast to depart from the group. This feature is useful in practice because robots may have to explore an environment while keeping sufficiently close to others to maintain wireless connections. In addition, the system is resilient to robot additions or removals. For example, if the connectivity of the swarm is broken due to the removal of some robots, the isolated ones that are already in the maze would stop moving. As more and more robots move into the maze, the isolated ones would get connected to the others again.

## Discussion

The distributed negotiation and control strategy proposed in this work is free of both centralized and decentralized goal assignment procedures. This strategy introduces a mean-shift exploration mechanism so that each robot can actively explore unoccupied locations around them to avoid inter-robot competition for the same locations. The mean-shift exploration is achieved through an adapted version of the mean-shift algorithm so that each robot keeps seeking the highest density of unoccupied cells. It is noticed that the mean-shift algorithm is also adopted in ref. ^25^ to achieve shape assembly. However, the mean-shift algorithm therein is used as an alternative to generating attractive and repulsive forces and hence is not able to resolve the inter-robot competition problem.

Experimental results verified that the proposed strategy can assemble highly nonconvex shapes and exhibits strong adaptability against mismatches between the number of robots and the number of cells in the desired shape. The strategy can also be applied to solve some challenging tasks such as cooperative cargo transportation and environment exploration with minimal modifications of the algorithms. It, therefore, provides a promising method for efficient and adaptive shape assembly tasks of robot swarms.

The desired shape in a shape assembly task is a global constraint for all the robots. The global constraint sets a fundamental requirement that certain global information must be used during the process of shape assembly. However, the required global information may exhibit in different forms in different methods. For example, in the proposed strategy and the state-of-the-art ones^10,12,30^, the required global information is that every robot has a common sense on a global reference frame. With this global information, every robot can evaluate its location relative to the desired shape. In practice, the global reference frame can be obtained by GPS in outdoor open spaces. If the global reference frame is unavailable, there must be alternatives to acquire global information. For example, the method in ref. ^18^ replaces the global reference frame by using a relative positioning system based on four seed robots.

The proposed negotiation and control algorithms are applied to assemble 2D shapes in this work. They can be extended to 3D scenarios since these algorithms are based on vector calculations where the position and velocity vectors are not restricted to 2D. However, a new human-swarm interface that can specify 3D shapes is required. In addition to user-specified geometric shapes, the proposed algorithms may be extended to assemble shapes specified in a natural way. For example, a swarm of robots may be used to clean an area of chemical pollutants in a water body. Here, the area of the densest pollutants can be treated as the desired shape and the density change in the nearby water can be regarded as the gradient. In this case, the shape is “specified” by the environment naturally and the proposed algorithms can be potentially applied.

One limitation of the proposed strategy is that it can only assemble shapes with single connected components. That is because the mean-shift exploration of each robot is based on local sensing of unoccupied locations. Since the sensing range of each robot is limited, if two disconnected shape components are far away from each other, it is difficult for a robot to explore unoccupied locations across different shape components. This problem may be potentially solved in several ways in practice. The first is to globally assign the robots to different components in advance. This way requires a centralized assignment process. A simple distributed solution is to add a line segment to bridge two disconnected components so that the entire shape becomes connected. In this way, each robot can explore the bridge to move from one shape component to another.

Moreover, we achieved the environment exploration tasks by treating them as shape assembly tasks, where the map of the environment is known and specified as the desired shape. While the map of the environment to be explored may not be available in practical search and rescue tasks, the proposed strategy can be applied if a minimal modification is made so that any regions unoccupied by obstacles are treated as desired shape regions to fulfill. Finally, although the algorithms proposed in this work are fully distributed, the physical robots in our experiments were provided data from a centralized motion capture system. It will be a promising research direction to develop robotic hardware systems with onboard sensing units to realize fully distributed shape assembly tasks of large-scale robot swarms.

## Methods

### Specify a desired shape as a black-white grid

The proposed human-swarm interface allows the user to either load a predesigned image or manually draw one in the workspace (Supplementary Figure 1). Either way, we can obtain a binary grid with black and white cells. Each cell of the grid is described by two basic parameters ***ρ*** and *ξ*_***ρ***_. Here, ***ρ*** = (*ρ*_*x*_, *ρ*_*y*_) is the column and row indexes of the cell and *ξ*_***ρ***_ ∈ {0, 1} represents the color of the cell: *ξ*_***ρ***_ = 0 if the cell is black and *ξ*_***ρ***_ = 1 if the cell is white. The desired shape corresponds to the set of black cells. More information is given in Section 1 in the Supplementary Information.

### Convert the desired shape to a grayscale grid

The purpose to convert the black-white grid to a grayscale grid is to expand the influence scope of the desired shape so that the robots can move into the desired shape more smoothly. The gray conversion is based on the distance transformation algorithm^29^. Specifically, we expand the set of black cells out by *h* cells to generate an *h*-level grayscale grid. The gray level of each cell denoted as *ξ*_***ρ***_ is calculated based on a local parallel method described in ref. ^29^. In particular, for any cell ***ρ*** in the grid, its gray value is calculated iteratively by

1 $${\xi }_{{{{{{{{\boldsymbol{\rho }}}}}}}}}^{k}=\mathop{\min }\limits_{{{{{{{{{\boldsymbol{\rho }}}}}}}}}^{{\prime} }\in {{{{{{{{\mathcal{M}}}}}}}}}_{{{{{{{{\boldsymbol{\rho }}}}}}}}}}\left({\xi }_{{{{{{{{{\boldsymbol{\rho }}}}}}}}}^{{\prime} }}^{k-1}+\frac{1}{h}\right),\quad k=1,\,2,\ldots,\,h-1.$$

Here, the superscript *k* denotes the *k*-th iteration. In every iteration, we conduct (1) for each cell. We need at most *h* − 1 iterations to obtain the final grayscale grid^29^. Here, ${{{{{{{{\mathcal{M}}}}}}}}}_{{{{{{{{\boldsymbol{\rho }}}}}}}}}$ is a 3 × 3 mask centered at the cell ***ρ***. Illustrative examples and more information can be found in Section 1 in the Supplementary Information.

### Parameterization of the size of each cell in the grid

The desired shape represented by a grid obtained from the human-swarm interface is merely graphical. Some important parameters of the desired shape are to be determined automatically. The first parameter is the size length of each cell denoted as *ℓ*_cell_. On the one hand, the total area of all the black cells is ${\ell }_{{{{{{{{\rm{cell}}}}}}}}}^{2}{n}_{{{{{{{{\rm{cell}}}}}}}}}$. On the other hand, let *r*_avoid_ be the collision avoidance distance between the centers of any two robots. It is expected that the distance among each pair of robots in the desired shape is equal to *r*_avoid_ (Supplementary Figure 2c). As a result, the space occupied by each robot could be approximated by a circle with the center at the robot and the radius as *r*_avoid_/2. Thus, the area occupied by all the *n*_robot_ robots is $\pi {({r}_{{{{{{{{\rm{avoid}}}}}}}}}/2)}^{2}{n}_{{{{{{{{\rm{robot}}}}}}}}}$. The above two areas are expected to be equal so that the robots could cover the desired shape (Supplementary Figure 2c):

$$\frac{\pi }{4}{r}_{{{{{{{{\rm{avoid}}}}}}}}}^{2}{n}_{{{{{{{{\rm{robot}}}}}}}}}\,\approx \,{\ell }_{{{{{{{{\rm{cell}}}}}}}}}^{2}{n}_{{{{{{{{\rm{cell}}}}}}}}}$$

from which *ℓ*_cell_ can be solved as

2 $${\ell }_{{{{{{{{\rm{cell}}}}}}}}}=\sqrt{\frac{\pi }{4}\frac{{n}_{{{{{{{{\rm{robot}}}}}}}}}}{{n}_{{{{{{{{\rm{cell}}}}}}}}}}}{r}_{{{{{{{{\rm{avoid}}}}}}}}}.$$

Equation (2) indicates that, when *n*_robot_ and *r*_avoid_ are given, *ℓ*_cell_ is inversely proportional to $\sqrt{{n}_{{{{{{{{\rm{cell}}}}}}}}}}$.

It is notable that *n*_cell_ is specified by the user when drawing a shape in the human-swarm interface. If *n*_cell_ = *n*_robot_, equation (2) becomes ${\ell }_{{{{{{{{\rm{cell}}}}}}}}}=\sqrt{\frac{\pi }{4}}{r}_{{{{{{{{\rm{avoid}}}}}}}}}\,\approx \,{r}_{{{{{{{{\rm{avoid}}}}}}}}}$, which means the side length of each cell is equal to the avoidance distance. In this case, each robot can approximately occupy one cell. However, our method does not necessarily require *n*_cell_ = *n*_robot_, which is a strict condition. The proposed method allows more general cases where *n*_cell_ and *n*_robot_ may mismatch (see examples in Fig. 5 and Supplementary Figure 5).

### Parameterization of the position and orientation of the desired shape

Another two parameters of the desired shape are the center position and orientation. There is no centralized assignment of the position or orientation. The robots can negotiate the two parameters in a distributed manner.

Before proceeding further, we need to introduce some necessary notations that will be used frequently later. Suppose there are *n*_robot_ mobile robots in ${{\mathbb{R}}}^{2}$ and *n*_robot_ ≥ 2. Each robot is regarded as a circle with the radius as *r*_body_. Let ${{{{{{{{\bf{p}}}}}}}}}_{i}\in {{\mathbb{R}}}^{2}$ be the position of the center point of robot *i* in a global coordinate frame. The dynamic model of each robot is assumed to be ${\dot{{{{{{{{\bf{p}}}}}}}}}}_{i}={{{{{{{{\bf{v}}}}}}}}}_{i}$ where *i* = 1, …, *n*_robot_. Here, **v**_*i*_ is the velocity command to be designed. When the distance between two robots is less than a threshold *r*_sense_, the two robots could share information with each other. The information network defines an undirected graph ${{{{{{{\mathcal{G}}}}}}}}=({{{{{{{\mathcal{V}}}}}}}},\,{{{{{{{\mathcal{E}}}}}}}})$, which consists of a vertex set ${{{{{{{\mathcal{V}}}}}}}}=\{1,\ldots,\,{n}_{{{{{{{{\rm{robot}}}}}}}}}\}$ and an edge set ${{{{{{{\mathcal{E}}}}}}}}\subseteq {{{{{{{\mathcal{V}}}}}}}}\times {{{{{{{\mathcal{V}}}}}}}}$ such that ${{{{{{{\mathcal{E}}}}}}}}=\{(i,\,j):\parallel {{{{{{{{\bf{p}}}}}}}}}_{i}-{{{{{{{{\bf{p}}}}}}}}}_{j}\parallel < \,{r}_{{{{{{{{\rm{sense}}}}}}}}},\,j\ne i\}$. Here, ∥ ⋅ ∥ is the Euclidean norm. The set of neighbors of robot *i* is ${{{{{{{{\mathcal{N}}}}}}}}}_{i}=\{j\in {{{{{{{\mathcal{V}}}}}}}}:(i,\,j)\in {{{{{{{\mathcal{E}}}}}}}}\}$.

Suppose ***ρ***_*o*_ is the cell located closest to the center of the desired shape. Let ${{{{{{{{\bf{p}}}}}}}}}_{{{{{{{{{\boldsymbol{\rho }}}}}}}}}_{o}}$ and ${{{{{{{{\bf{v}}}}}}}}}_{{{{{{{{{\boldsymbol{\rho }}}}}}}}}_{o}}$ be the position and velocity of the center point of cell ***ρ***_*o*_ in a global reference frame. Let *ϕ* denote the orientation angle of the desired shape. Then, the position and orientation of the desired shape could be represented by ${{{{{{{{\bf{p}}}}}}}}}_{{{{{{{{{\boldsymbol{\rho }}}}}}}}}_{o}}$ and *ϕ*, respectively.

Every robot has its own interpretation of ${{{{{{{{\bf{p}}}}}}}}}_{{{{{{{{{\boldsymbol{\rho }}}}}}}}}_{o}}$ and ${{{{{{{{\bf{v}}}}}}}}}_{{{{{{{{{\boldsymbol{\rho }}}}}}}}}_{o}}$, denoted as ${{{{{{{{\bf{p}}}}}}}}}_{{{{{{{{{\boldsymbol{\rho }}}}}}}}}_{o},i}$ and ${{{{{{{{\bf{v}}}}}}}}}_{{{{{{{{{\boldsymbol{\rho }}}}}}}}}_{o},i}$. Initially, ${{{{{{{{\bf{p}}}}}}}}}_{{{{{{{{{\boldsymbol{\rho }}}}}}}}}_{o},i}({t}_{0})={{{{{{{{\bf{p}}}}}}}}}_{i}({t}_{0})$ and ${{{{{{{{\bf{v}}}}}}}}}_{{{{{{{{{\boldsymbol{\rho }}}}}}}}}_{o},i}({t}_{0})={{{{{{{{\bf{v}}}}}}}}}_{i}({t}_{0})$, which means that each robot initially treats itself as the center of the desired group shape. The interpretations of different robots can gradually reach a consensus by the following distributed negotiation algorithm:

3 $${{{{{{{{\bf{v}}}}}}}}}_{{{{{{{{{\boldsymbol{\rho }}}}}}}}}_{o},i}=-\frac{{c}_{1}}{|{{{{{{{{\mathcal{N}}}}}}}}}_{i}|}\mathop{\sum}\limits_{j\in {{{{{{{{\mathcal{N}}}}}}}}}_{i}}{{{{{{{\rm{sign}}}}}}}}({{{{{{{{\bf{p}}}}}}}}}_{{{{{{{{{\boldsymbol{\rho }}}}}}}}}_{o},i}-{{{{{{{{\bf{p}}}}}}}}}_{{{{{{{{{\boldsymbol{\rho }}}}}}}}}_{o},j})|{{{{{{{{\bf{p}}}}}}}}}_{{{{{{{{{\boldsymbol{\rho }}}}}}}}}_{o},i}-{{{{{{{{\bf{p}}}}}}}}}_{{{{{{{{{\boldsymbol{\rho }}}}}}}}}_{o},j}{|}^{\alpha }+\frac{1}{|{{{{{{{{\mathcal{N}}}}}}}}}_{i}|}\mathop{\sum}\limits_{j\in {{{{{{{{\mathcal{N}}}}}}}}}_{i}}{{{{{{{{\bf{v}}}}}}}}}_{{{{{{{{{\boldsymbol{\rho }}}}}}}}}_{o},j}.$$

There are two terms in (3). The first term in (3) is the average of the deficiency of the position interpretations between robot *i* and its neighbors. Here, sign( ⋅ ) and ∣ ⋅ ∣ denote the sign and the absolute value of a real number. In addition, *c*_1_ > 0 and 0 < *α* < 1 are two constant gains. The role of the first term is to drive ${{{{{{{{\bf{p}}}}}}}}}_{{{{{{{{{\boldsymbol{\rho }}}}}}}}}_{o},i}\to {{{{{{{{\bf{p}}}}}}}}}_{{{{{{{{{\boldsymbol{\rho }}}}}}}}}_{o},j}$ where $j\in {{{{{{{{\mathcal{N}}}}}}}}}_{i}$. The second term is the average of the velocities of the neighboring robots. Its role is to drive ${{{{{{{{\bf{v}}}}}}}}}_{{{{{{{{{\boldsymbol{\rho }}}}}}}}}_{o},i}\to {{{{{{{{\bf{v}}}}}}}}}_{{{{{{{{{\boldsymbol{\rho }}}}}}}}}_{o},j}$ where $j\in {{{{{{{{\mathcal{N}}}}}}}}}_{i}$. Since *α* < 1, consensus can be achieved in a finite time. This is important for speeding up the negotiation process. The convergence analysis is given in Theorem 1 in the Supplementary Information.

Regarding the orientation negotiation, let *ϕ*_*i*_ be the interpretation of *ϕ* by robot *i*. The initial values of *ϕ*_*i*_ could be randomly selected or based on task-oriented requirements. The distributed orientation negotiation algorithm is

4 $${\dot{\phi }}_{i}=-\frac{{c}_{2}}{|{{{{{{{{\mathcal{N}}}}}}}}}_{i}|}\mathop{\sum}\limits_{j\in {{{{{{{{\mathcal{N}}}}}}}}}_{i}}{{{{{{{\rm{sign}}}}}}}}({\phi }_{i}-{\phi }_{j})|{\phi }_{i}-{\phi }_{j}{|}^{\alpha }+\frac{1}{|{{{{{{{{\mathcal{N}}}}}}}}}_{i}|}\mathop{\sum}\limits_{j\in {{{{{{{{\mathcal{N}}}}}}}}}_{i}}{\dot{\phi }}_{j}$$

where *c*_2_ is a positive constant coefficient. This algorithm has the same structure as (3) and can be analyzed analogously.

If the desired shape is required to track a specified trajectory, we can introduce informed robots to achieve that. Details are given in Section 2 in Supplementary Information.

### Shape-entering velocity command

The shape-entering velocity command ${{{{{{{{\bf{v}}}}}}}}}_{i}^{{{{{{{{\rm{ent}}}}}}}}}$ in (5) aims to drive robot *i* into its interpretation of the desired shape, as depicted in Supplementary Figure 3a. In particular, it is designed as

5 $${{{{{{{{\bf{v}}}}}}}}}_{i}^{{{{{{{{\rm{ent}}}}}}}}}={\kappa }_{1}{\xi }_{{{{{{{{{\boldsymbol{\rho }}}}}}}}}_{i}}\frac{{{{{{{{{\bf{p}}}}}}}}}_{{{{{{{{\rm{T}}}}}}}},i}-{{{{{{{{\bf{p}}}}}}}}}_{i}}{\parallel {{{{{{{{\bf{p}}}}}}}}}_{{{{{{{{\rm{T}}}}}}}},i}-{{{{{{{{\bf{p}}}}}}}}}_{i}\parallel }+{{{{{{{{\bf{v}}}}}}}}}_{{{{{{{{{\boldsymbol{\rho }}}}}}}}}_{o},i}$$

where **p**_*i*_ is the position of robot *i* and **p**_T,*i*_ is a target location for robot *i* to move toward. Hence, $\frac{{{{{{{{{\bf{p}}}}}}}}}_{{{{{{{{\rm{T}}}}}}}},i}-{{{{{{{{\bf{p}}}}}}}}}_{i}}{\parallel {{{{{{{{\bf{p}}}}}}}}}_{{{{{{{{\rm{T}}}}}}}},i}-{{{{{{{{\bf{p}}}}}}}}}_{i}\parallel }$ is a unit vector pointing from **p**_*i*_ to **p**_T,*i*_. Therefore, the first term in (5) drives the robot toward its target location. Here, ${\kappa }_{1}{\xi }_{{{{{{{{{\boldsymbol{\rho }}}}}}}}}_{i}}$ is a control gain where ***ρ***_*i*_ is the index of the cell that is closest to ${{{{{{{{\bf{p}}}}}}}}}_{i},\,{\xi }_{{{{{{{{{\boldsymbol{\rho }}}}}}}}}_{i}}$ is the gray level of that cell, and *κ*_1_ > 0 is a constant gain. The second term ${{{{{{{{\bf{v}}}}}}}}}_{{{{{{{{{\boldsymbol{\rho }}}}}}}}}_{o},i}$ in (5) is the local interpretation of robot *i* on the moving velocity of the entire shape. This term is necessary when the desired shape is moving. The calculation of the cell index ***ρ***_*i*_ and the target location **p**_T,*i*_ is given in Section 3 in the Supplementary Information.

### Shape-exploration velocity command

The shape-exploration velocity command ${{{{{{{{\bf{v}}}}}}}}}_{i}^{\exp }$ in (6) aims to push robot *i* into the desired shape and explore unoccupied black cells. Here, a cell is defined as occupied if the distance between its center and any robot is less than *r*_avoid_/2; and unoccupied otherwise.

In particular, we employ the mean-shift concept^26^ to design ${{{{{{{{\bf{v}}}}}}}}}_{i}^{\exp }$ as

6 $${{{{{{{{\bf{v}}}}}}}}}_{i}^{\exp }=\frac{{\sum }_{{{{{{{{\boldsymbol{\rho }}}}}}}}\in {{{{{{{{\mathcal{M}}}}}}}}}_{i}^{{{{{{{{\rm{neigh}}}}}}}}}}{\kappa }_{2}\psi (\parallel {{{{{{{{\bf{p}}}}}}}}}_{{{{{{{{\boldsymbol{\rho }}}}}}}}}-{{{{{{{{\bf{p}}}}}}}}}_{i}\parallel /{r}_{{{{{{{{\rm{sense}}}}}}}}})\left({{{{{{{{\bf{p}}}}}}}}}_{{{{{{{{\boldsymbol{\rho }}}}}}}}}-{{{{{{{{\bf{p}}}}}}}}}_{i}\right)}{{\sum }_{{{{{{{{\boldsymbol{\rho }}}}}}}}\in {{{{{{{{\mathcal{M}}}}}}}}}_{i}^{{{{{{{{\rm{neigh}}}}}}}}}}\psi (\parallel {{{{{{{{\bf{p}}}}}}}}}_{{{{{{{{\boldsymbol{\rho }}}}}}}}}-{{{{{{{{\bf{p}}}}}}}}}_{i}\parallel /{r}_{{{{{{{{\rm{sense}}}}}}}}})}$$

which is a normalized weighted average of **p**_***ρ***_ − **p**_*i*_, where **p**_*i*_ is robot *i*’s position and **p**_***ρ***_ is a position of a valid cell whose index is ***ρ***. Here, ***ρ*** belongs to the set ${{{{{{{{\mathcal{M}}}}}}}}}_{i}^{{{{{{{{\rm{neigh}}}}}}}}}$. The factor *κ*_2_ ∈ {*σ*_1_, *σ*_2_} with *σ*_1_, *σ*_2_ > 0 is a positive constant. The weight for **p**_***ρ***_ − **p**_*i*_ is a function *ψ* defined as

$$\psi (z)=\left\{\begin{array}{ll}1,\hfill&z\le 0\\ \frac{1}{2}(1+\cos \pi z),&0 \, < \,z \, < \,1\\ 0,\hfill&z\ge 1\end{array}\right..$$

This function is monotonically decreasing from 1 to 0 as *z* increases. As a result, the weight *ψ*(∥**p**_***ρ***_ − **p**_*i*_∥/*r*_sense_) is large when the distance between **p**_***ρ***_ and **p**_*i*_ is small. Hence, more weights are given to the cells that are closer to robot *i*.

The velocity command in (6) encourages robot *i* to explore unoccupied black cells. Here, ${{{{{{{{\mathcal{M}}}}}}}}}_{i}^{{{{{{{{\rm{neigh}}}}}}}}}$ is defined as the set of all the unoccupied black cells that are within the sensing radius *r*_sense_ of the robot (see Supplementary Figure 3b) and *κ*_2_ = *σ*_2_. In addition, when robot *i* is close to the boundary of the shape so that there are non-black cells within the sensing radius, on top of exploring unoccupied black cells, the velocity command also aims to push robot *i* into the desired shape. In this case, ${{{{{{{{\mathcal{M}}}}}}}}}_{i}^{{{{{{{{\rm{neigh}}}}}}}}}$ includes all the black cells no matter whether they are occupied or not and *κ*_2_ = *σ*_1_.

### Interaction velocity command

The aim of the interaction velocity command ${{{{{{{{\bf{v}}}}}}}}}_{i}^{{{{{{{{\rm{int}}}}}}}}}$ in (7) is to achieve collision avoidance and velocity alignment. To that end, it is designed as

7 $${{{{{{{\mathbf{v}}}}}}}}_{i}^{{{{{{{\rm{int}}}}}}}}=\underbrace{\kappa_3 \mathop{\sum}\limits_{j \in {{{{{{\mathcal{N}}}}}}}_i \cup {{{{{{\mathcal{O}}}}}}}_i}{\mu(||{{{{{{{\mathbf{p}}}}}}}}_i-{{{{{{{\mathbf{p}}}}}}}}_j||) \left({{{{{{{\mathbf{p}}}}}}}}_i-{{{{{{{\mathbf{p}}}}}}}}_j\right)}}_{{{{{{{{\mathrm{first}}}}}}}\,\,{{{{{{\mathrm{term}}}}}}}}} - \underbrace{\mathop{\sum}\limits_{j \in {{{{{{\mathcal{N}}}}}}}_i}{\frac{1}{|{{{{{{\mathcal{N}}}}}}}_i|} \left({{{{{{{\mathbf{v}}}}}}}}_i-{{{{{{{\mathbf{v}}}}}}}}_j\right)}}_{{{{{{{{\mathrm{second}}}}}}}\,\,{{{{{{\mathrm{term}}}}}}}}}$$

where *κ*_3_ is a positive control gain.

The first term in (7) is a weighted sum of **p**_*i*_ − **p**_*j*_, where **p**_*i*_ is the position of robot *i* and **p**_*j*_ is the position of the neighboring robot *j* or a collision point *j*. Here, $j\in {{{{{{{{\mathcal{N}}}}}}}}}_{i}\cup {{{{{{{{\mathcal{O}}}}}}}}}_{i}$ where ${{{{{{{{\mathcal{N}}}}}}}}}_{i}$ is the set of neighboring robots and ${{{{{{{{\mathcal{O}}}}}}}}}_{i}$ is the set of collision points (see Supplementary Figure 3c). The weight *μ*(∥**p**_*i*_ − **p**_*j*_∥) is defined as

$$\mu (\parallel {{{{{{{{\bf{p}}}}}}}}}_{i}-{{{{{{{{\bf{p}}}}}}}}}_{j}\parallel )=\left\{\begin{array}{ll}\frac{{r}_{{{{{{{{\rm{avoid}}}}}}}}}}{\parallel {{{{{{{{\bf{p}}}}}}}}}_{i}-{{{{{{{{\bf{p}}}}}}}}}_{j}\parallel }-1,&\parallel {{{{{{{{\bf{p}}}}}}}}}_{i}-{{{{{{{{\bf{p}}}}}}}}}_{j}\parallel \le {r}_{{{{{{{{\rm{avoid}}}}}}}}}\\ 0,\hfill&\parallel {{{{{{{{\bf{p}}}}}}}}}_{i}-{{{{{{{{\bf{p}}}}}}}}}_{j}\parallel > \,{r}_{{{{{{{{\rm{avoid}}}}}}}}}\end{array}\right..$$

By definition, when ∥**p**_*i*_ − **p**_*j*_∥ is close to zero, the weight *μ*(∥**p**_*i*_ − **p**_*j*_∥) methods to infinity. When ∥**p**_*i*_ − **p**_*j*_∥ is close to *r*_avoid_, the weight *μ*(∥**p**_*i*_ − **p**_*j*_∥) monotonically decreases to zero. Since **p**_*i*_ − **p**_*j*_ is a vector pointing from **p**_*j*_ to **p**_*i*_, the first term is a repulsive velocity that pushes robot *i* away from its surroundings to avoid a collision. The second term in (7) aims to align robot *i*’s velocity with its neighbors. Velocity alignment can help reduce the chance of inter-robot collision. In the meantime, it is necessary when the entire shape needs to track a moving trajectory.

### Performance metrics

To evaluate the performance of the proposed strategy, we consider the following four metrics.

The first metric, coverage rate, is to evaluate the proportion of the black cells in the desired shape that are occupied by robots. Here, a cell is defined as occupied if the distance between its center and any robot is less than *r*_avoid_/2. The metric is defined as

$${M}_{1}=\frac{{n}_{{{{{{{{\rm{occ}}}}}}}}}}{{n}_{{{{{{{{\rm{cell}}}}}}}}}}\times 100\%$$

where *n*_occ_ is the number of black cells occupied by robots. If all black cells are occupied, that is *n*_occ_ = *n*_cell_, then *M*_1_ = 100%. If no black cells are occupied, then *M*_1_ = 0.

The second metric, entering rate, is the proportion of the robots that have entered the desired shape. Here, a robot is said to be inside the shape if it occupies a black cell. The metric is defined as

$${M}_{2}=\frac{{n}_{{{{{{{{\rm{in}}}}}}}}}}{{n}_{{{{{{{{\rm{robot}}}}}}}}}}\times 100\%$$

where *n*_in_ is the number of robots inside the desired shape. If all robots are inside the desired shape, then *M*_2_ = 100%, and otherwise *M*_2_ < 100%.

The third metric, distribution uniformity, measures the distribution uniformity of the robots in the desired shape. Denote ${r}_{\min,i}={\min }_{j\in {{{{{{{{\mathcal{N}}}}}}}}}_{i}}\parallel {{{{{{{{\bf{p}}}}}}}}}_{i}-{{{{{{{{\bf{p}}}}}}}}}_{j}\parallel$ as the minimum distance from robot *i* to its neighbors. Then, the metric is defined as

$${M}_{3}=\mathop{\sum }\limits_{i=1}^{n}{({r}_{\min,i}-{\bar{r}}_{\min,i})}^{2}$$

where ${\bar{r}}_{\min,i}=\frac{1}{n}\mathop{\sum }\nolimits_{i=1}^{n}{r}_{\min \!,i}$. If the distance between every pair of robots is the same, then *M*_3_ = 0; otherwise, *M*_3_ > 0.

The fourth metric, velocity polarization, is to measure the polarization of the velocities of the robots. The metric is defined as:

$${M}_{4}=\frac{\left|\mathop{\sum }\nolimits_{i=1}^{n}{{{{{{{{\bf{v}}}}}}}}}_{i}\right|}{\mathop{\sum }\nolimits_{i=1}^{n}\left|{{{{{{{{\bf{v}}}}}}}}}_{i}\right|}.$$

If all robots move with the same velocity, then *M*_4_ = 1. If the robots’ velocities are random, then *M*_4_ is close to zero.

### Statistics and reproducibility

Data analysis was done by using the native functions of Originlab. All the statistical results are displayed in the format of “average ± minimum/maximum”. During the statistical analysis, no statistical method was used to predetermine sample size, and no data were excluded. All statistical trials used random initialization of robots’ locations and their interpretations of the desired shape. In all bar charts, the lower and upper edges of the bar represent the minimum and maximum, and the shaded region represents the average.

We implemented the simulation in Matlab. Details of the simulation setup and parameters used are given in Section 4 in the Supplementary Information. In our experiments, we use 50 holonomic wheeled robots. We implemented the experiments in Visual Studio using C++. Details of the experimental setup, robotic platform, and parameter settings are given in Section 5 in the Supplementary Information.

## Supplementary information

Supplementary Information

Description to Additional Supplementary Information

Supplementary Movie 1

Supplementary Movie 2

Supplementary Movie 3

Supplementary Movie 4

Supplementary Movie 5

Supplementary Movie 6

## Data Availability

The data used in this study are available from the corresponding author upon request.

## Code availability

The code of the human-swarm interface used in this study is publicly available at https://github.com/WestlakeAerialRobotics/Human-swarm-interface(DOI: 10.5281/zenodo.7960508).
