# Supplementary material for: Mean-shift exploration in shape assembly of robot swarms
Source: Nat Commun. 2023 Jun 13;14:3476. doi: 10.1038/s41467-023-39251-5 (PMC10264375; doi:10.1038/s41467-023-39251-5)
Supplement: Supplementary file 1 — Supplementary Information [file 41467_2023_39251_MOESM1_ESM.pdf]

Supplementary Information for

Mean-shift exploration in shape assembly of robot swarms

Guibin Sun *et al.*

This PDF file includes:

Supplementary Methods

Supplementary Figures 1 to 12

Supplementary Tables 1 to 3

Supplementary References

Other supplementary material for this manuscript includes the following:

Supplementary Movies 1 to 6

Supplementary Movie 1: Complex shape assembly - Snowflake shape assembly

Supplementary Movie 2: Complex shape assembly - Letters “ROBOT” assembly

Supplementary Movie 3: Adaptability to swarm scale changes

Supplementary Movie 4: Cooperative cargo transportation

Supplementary Movie 5: Environment exploration - Entering a passenger elevator

Supplementary Movie 6: Environment exploration - Exploring a complex maze

Supplementary Methods

1 Additional Information of Human-Swarm Interface

This section provides more information about the human-swarm interface. The interface is shown in Supplementary Figure 1. It is coded by Matlab (R2020a). The code is available at https://github.com/WestlakeAerialRobotics/Human-swarm-interface.

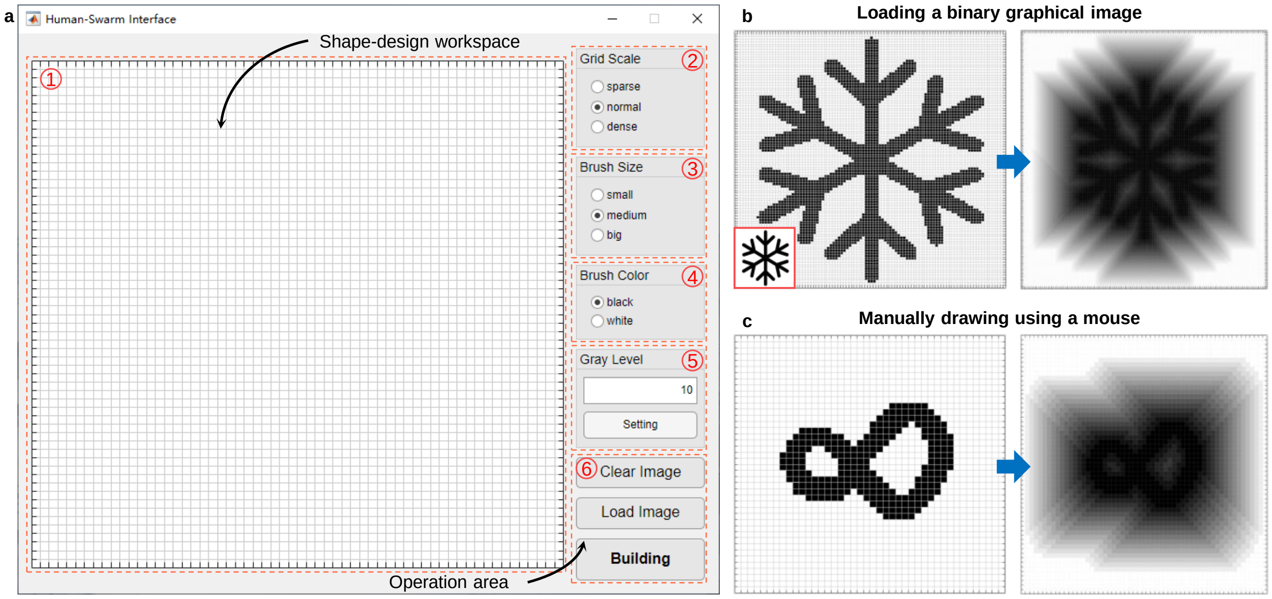

Supplementary Figure 1: The human-swarm interface. **a** A snapshot of the interface. There are six functional components: shape-design workspace, grid size setting of the workspace, grayscale setting for gray conversion, brush size selection, brush color selection, and operation area. **b** An example of specifying the desired shape by loading a predesigned binary image. The original image is shown in the bottom left. **c** An example of drawing a shape in the workspace using the brush tool.

After the human operator has designed a black-white grid, the next step is to convert the black-white grid to a grayscale one. Here, we present two examples to illustrate the iterative process described by the algorithm in (1) in the main text. First, consider the left grid in Supplementary Figure 2a. In this binary grid, there is only a single black cell. We set $h = 3$. After applying the iterative algorithm (1) in the main text to each cell of the left grid, we obtain the grayscale grid in the middle. After applying the algorithm to each cell in the middle grid, the right grayscale grid is generated. Second, Supplementary Figure 2b shows a grayscale grid generated from a rectangular shape given in Supplementary Figure 2c. The value of h is set to be 5 in this example. As we can see, when h increases, the gray area surrounding the black area enlarges.

How to select the value of h? If we select a greater value of h, the influence scope of the desired shape becomes larger. In this case, the gray level varies smoothly across neighboring cells. As a

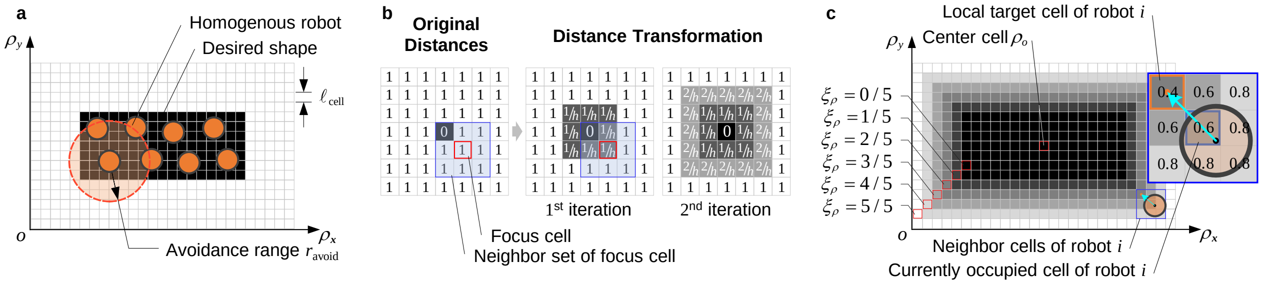

Supplementary Figure 2: Illustration of the gray conversion algorithm in (1) in the main text. **a** An example to demonstrate the iterative algorithm in (1) in the main text. **b** Another example to illustrate the grayscale grid obtained from a binary grid. **c** A rectangular desired shape and some notations.

result, the control command, which relies on the gray levels of the cells, changes smoothly. However, a greater value of h is also more computationally expensive. One heuristic value of h suggested by our extensive experiments is $h = \lceil \sqrt{n_{\text{cell}}}/2 \rceil$, where n_{cell} is the number of black cells and $\lceil \cdot \rceil$ denotes round up. In the simple case where the desired shape is a square, the heuristic value of h is half of the side length of the square.

After the grayscale grid has been generated, it is then sent to all the robots and stored in each robot's memory. For all the shapes used in our work, the number of cells varies from 1,443 to 76,629. The memory size of a grayscale grid stored as a mat file, Matlab's standard data format, varies from 0.72 KB to 94.4 KB, which are affordable for mainstream embedded computers nowadays. In particular, there are 76,629 cells in Supplementary Figure 4a, and the corresponding size is 94.4 KB. In Supplementary Figure 5b, there are 1,833 cells and the size is 0.81 KB.

In our method, all the cells in the desired shape are black and hence there is no gradient inside the shape. In this case, the exploration control command plays an important role in successfully assembling the shape. One may wonder if gradients can also be introduced inside the desired shape to better guide the robots. The reason that we do not adopt this way is that it usually has a strict requirement for the initial configuration and the robots may easily get trapped in local minima when the shape is complex.

2 Additional Information of the Negotiation Algorithms

To introduce a user-specified trajectory for the entire desired formation, we introduce a small number of *informed* robots, which know the trajectory reference. The basic idea is that the informed robots would insist on their interpretations of the desired shape during the negotiation process. As a result, the interpretations of the other uninformed ones would converge to those of the informed ones.

In particular, the uninformed robots still execute the algorithm in (3) in the main text. The in-

formed robots execute the following algorithm instead:

$$\mathbf{v}_{\boldsymbol{\rho}_o,i} = -\frac{c_1}{|\mathcal{N}_i|}\sum_{j\in\mathcal{N}_i}\text{sign}(\mathbf{p}_{\boldsymbol{\rho}_o,i}-\mathbf{p}_{\boldsymbol{\rho}_o,j})|\mathbf{p}_{\boldsymbol{\rho}_o,i}-\mathbf{p}_{\boldsymbol{\rho}_o,j}|^{\alpha} + c_3(\mathbf{p}_{\text{ref}}-\mathbf{p}_{\boldsymbol{\rho}_o,i}) + \mathbf{v}_{\text{ref}} \tag{1}$$

where c_3 is a positive constant. Here, $\mathbf{p}_{\text{ref}}, \mathbf{v}_{\text{ref}} \in \mathbb{R}^2$ are the position and velocity of a time-varying reference for the shape center. It is notable that the information of the reference is merely known by the informed robots.

Regarding the orientation negotiation, the uninformed robots still execute the algorithm in (4) in the main text. The informed robots execute the following algorithm:

$$\dot{\phi}_i = -\frac{c_2}{|\mathcal{N}_i|}\sum_{j\in\mathcal{N}_i}\text{sign}(\phi_i-\phi_j)|\phi_i-\phi_j|^{\alpha} + c_4(\phi_{\text{ref}}-\phi_i) + \dot{\phi}_{\text{ref}} \tag{2}$$

where c_4 is a positive constant coefficient. Here, $\phi_{\text{ref}} \in \mathbb{R}$ is a time-varying orientation angle that the desired shape should track. It is only known by the informed robots.

Using informed robots is a common method to introduce time-varying references to a multi-agent system in the fields of multi-agent consensus [1, 2] and formation control [3, 4]. In these fields, the informed/uninformed robots are often called leader/follower. When a swarm needs to follow a desired trajectory, the information about the desired trajectory must be conveyed to the swarm. One way to do so is to send the information directly to every robot. Another way that is more practical is to send the information to a small number of informed robots so that the information can then spread in the swarm via local interactions. In practice, an informed robot may have additional resources to communicate with a supervisor such as a human operator to acquire the desired trajectory. Or, it can behave autonomously based on advanced onboard sensing and decision-making systems to sense the current situation and then decide where to move.

3 Additional Information of the Control Algorithms

3.1 Self-localization: How to calculate $\boldsymbol{\rho}_i$

For the control command in (5) and (6) in the main text, we need to calculate the cell index $\boldsymbol{\rho}_i$. We next show how to do that. Since $\boldsymbol{\rho}_i$ is the index of the cell that is closest to robot i, calculating $\boldsymbol{\rho}_i$ is to self-localize robot i in the grid. To obtain $\boldsymbol{\rho}_i$, we first need to transform the spatial position $\mathbf{p}_i$ into a local frame attached to the desired shape. Recall that the translation and orientation of the desired shape are described by $\mathbf{p}_{\boldsymbol{\rho}_o}$ and ϕ, respectively. Define a local frame whose origin is located at $\mathbf{p}_{\boldsymbol{\rho}_o}$

and y-axis is aligned with ϕ. While $\mathbf{p}_i$ is the position of robot i in the global frame, the corresponding position in the local frame is

$$\mathbf{p}^{(o)}_{\boldsymbol{\rho}_i} = R^{-1}_{\phi_i}(\mathbf{p}_i - \mathbf{p}_{\boldsymbol{\rho}_o,i})$$

where R_{ϕ_i} is a rotation matrix defined as

$$R_{\phi_i} = \begin{bmatrix} \cos\phi_i & -\sin\phi_i \\ \sin\phi_i & \cos\phi_i \end{bmatrix}.$$

Then the cell that is closest to robot i can be calculated by

$$\boldsymbol{\rho}_i = \left\lfloor \frac{\mathbf{p}^{(o)}_{\boldsymbol{\rho}_i}}{\ell_{\text{cell}}} \right\rceil + \boldsymbol{\rho}_o$$

where operator $\lfloor\cdot\rceil$ means rounding to the nearest integer.

3.2 Local target position: How to calculate $\mathbf{p}_{\mathrm{T},i}$

For the control command in (5) in the main text, we need to calculate the cell index $\boldsymbol{\rho}_{\mathrm{T},i}$. To do that, we need to consider two cases. While $\boldsymbol{\rho}_i$ is the cell closest to robot i, if $\boldsymbol{\rho}_i$ is outside the grayscale desired grid, then robot i can select the nearest gray cell as the target cell $\boldsymbol{\rho}_{\mathrm{T},i}$. If $\boldsymbol{\rho}_i$ is inside the grayscale desired grid, robot i can select a cell with the smallest gray level inside a 3×3 mask centered at $\boldsymbol{\rho}_i$ as the target cell $\boldsymbol{\rho}_{\mathrm{T},i}$. Once the index of the target cell is obtained, the position of that cell can be calculated by

$$\mathbf{p}_{\mathrm{T},i} = R_{\phi_i}(\boldsymbol{\rho}_{\mathrm{T},i} - \boldsymbol{\rho}_o)\ell_{\text{cell}} + \mathbf{p}_{\boldsymbol{\rho}_o,i}. \tag{3}$$

See Supplementary Figure 2b for illustration.

Finally, an intuitive illustration of the proposed strategy is given in Supplementary Figure 3.

4 Additional Simulation Examples

In addition to the experimental and simulation results shown in the main text, we present other simulation examples in this section to further verify the proposed method.

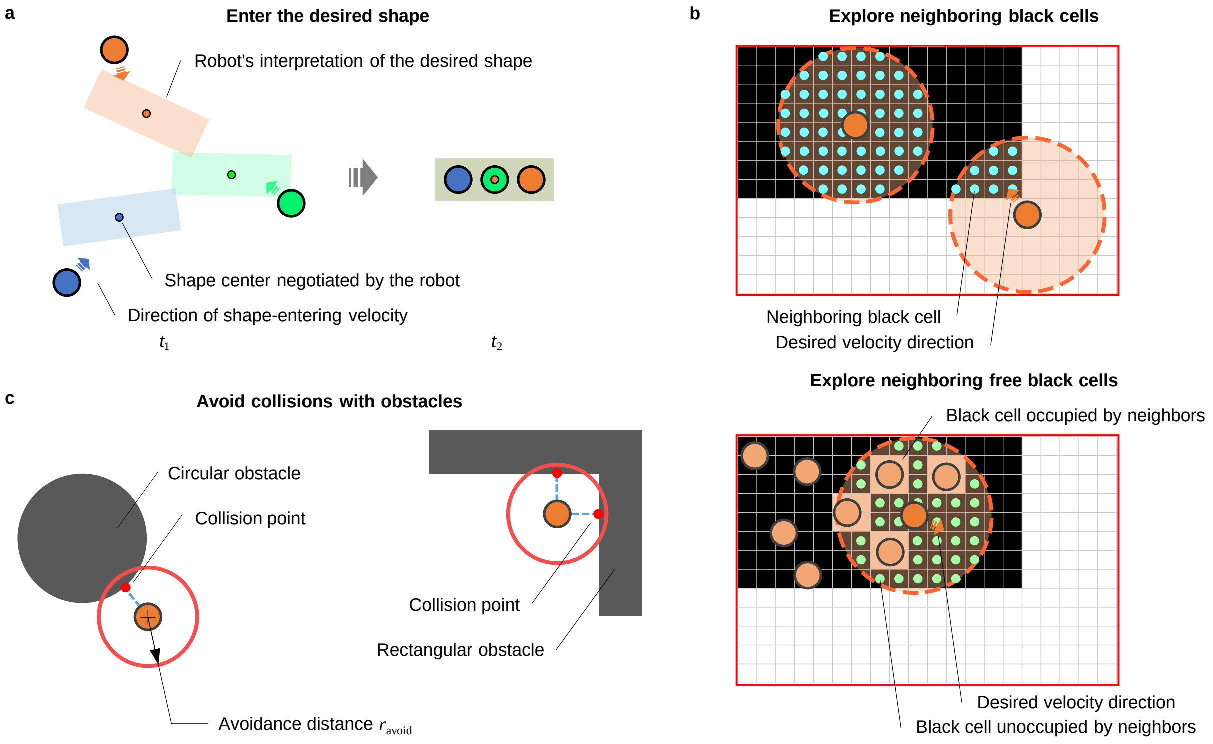

Supplementary Figure 3: Illustration of the three velocity commands in the main text. **a** An example to illustrate the principle of shape-entering velocity $\mathbf{v}_i^{\text{ent}}$. **b** Two examples to illustrate the shape-exploration velocity $\mathbf{v}_i^{\text{exp}}$. **c** wo examples to demonstrate obstacle avoidance by $\mathbf{v}_i^{\text{int}}$.

Supplementary Table 1: Simulation parameters.

Simulation examples		Parameters						
		κ_1	σ_1	σ_2	κ_3	α	c_1	c_2
Example 1	Supplementary Figure 4	10.0	10.0	20.0	25.0	0.8	1.6	1.6
Example 2	Supplementary Figure 5a	10.0	5.0	15.0	20.0	0.8	1.6	1.6
	Supplementary Figure 5b	10.0	5.0	15.0	25.0	0.8	1.6	1.6
	Supplementary Figure 5c	10.0	5.0	15.0	25.0	0.8	1.6	1.6
Example 3	Supplementary Figure 6	10.0	5.0	15.0	30.0	0.8	1.6	1.6
Example 4	Supplementary Figure 7	10.0	10.0	17.0	25.0	0.8	1.6	1.6

4.1 Simulation setup

In the simulation, each robot is modeled as a circular omnidirectional robot with a body size of $r_{\text{body}} = 0.2$ m. Each robot can sense or communicate with all the robots that lie within its sensing range $r_{\text{sense}} = 2.5$ m. Furthermore, collision avoidance is triggered if the inter-robot distance is less than $r_{\text{avoid}} = 1.5$ m. Other parameters for the following examples are listed in Supplementary Table 1.

4.2 Simulation examples

4.2.1 Example 1: Complex shapes

This example aims to further verify the assembly ability of the proposed method for more complex shapes. As shown in Supplementary Figure 4, the proposed method can successfully assemble planar versions of double helix structures of DNA. These shapes are highly nonconvex and asymmetrical. They are believed to be more challenging to assemble than the snowflake shape because they have many holes and thin branches.

4.2.2 Example 2: Adaptability

While it is favorable when n_{cell} matches n_{robot} so that each robot can occupy approximately one black cell, we challenge the proposed method by considering scenarios where n_{cell} and n_{robot} mismatch.

Supplementary Figure 5 shows 9 scenarios of assembling different desired shapes given different values of n_{cell} and n_{robot}. The value of n_{cell} varies from 170 to 2778. The value of n_{robot} varies from

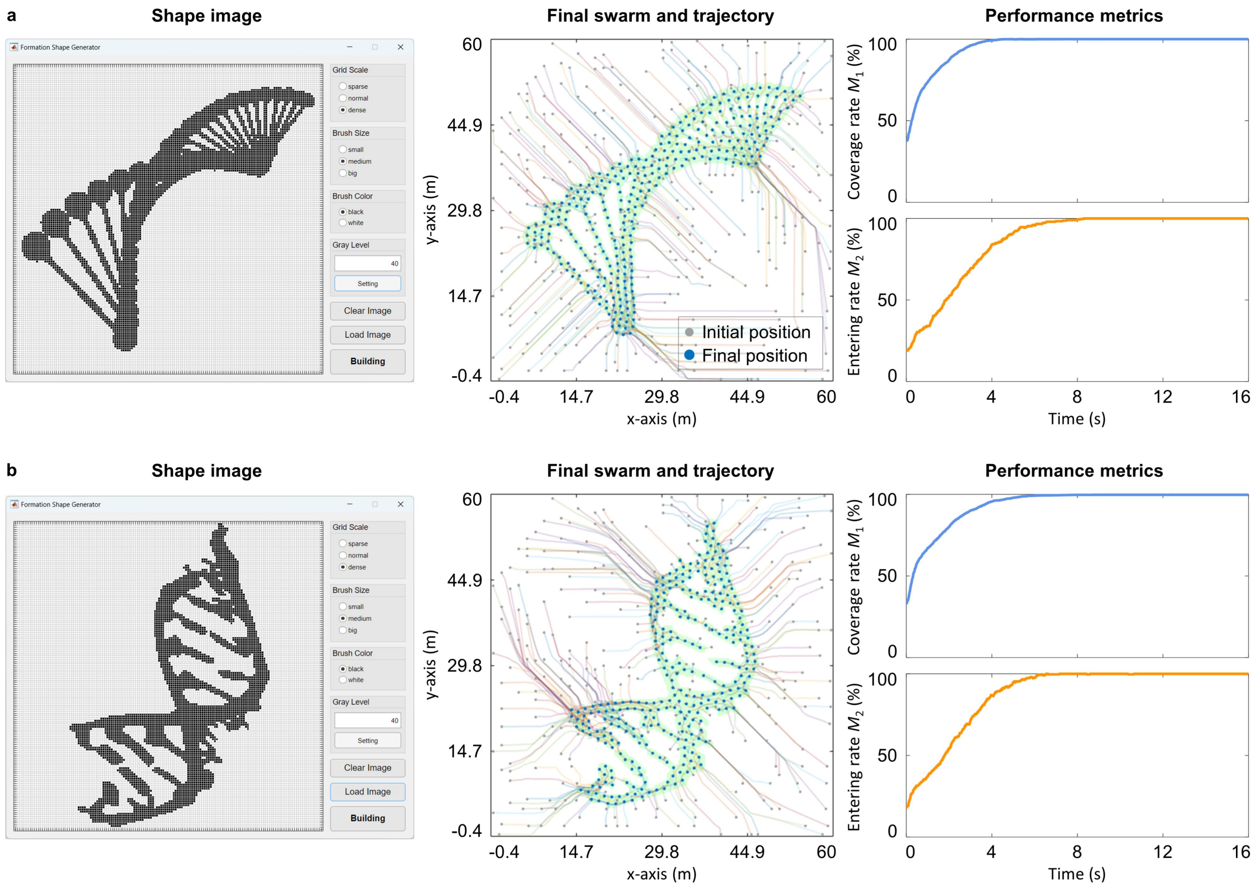

Supplementary Figure 4: Assembly of planar versions of double helix structures of DNA by the proposed method. The shape images in (A) and (B) are inspired by [5] and [6], respectively. The swarm has 300 robots.

16 to 256. Each column of the 9 subfigures has the same value of n_{robot}. Each row has the same n_{cell} and the same desired shape. The physical size of each cell is calculated adaptively by (2) in the main text.

In these scenarios, the desired shapes can be well assembled even though n_{cell} and n_{robot} mismatch

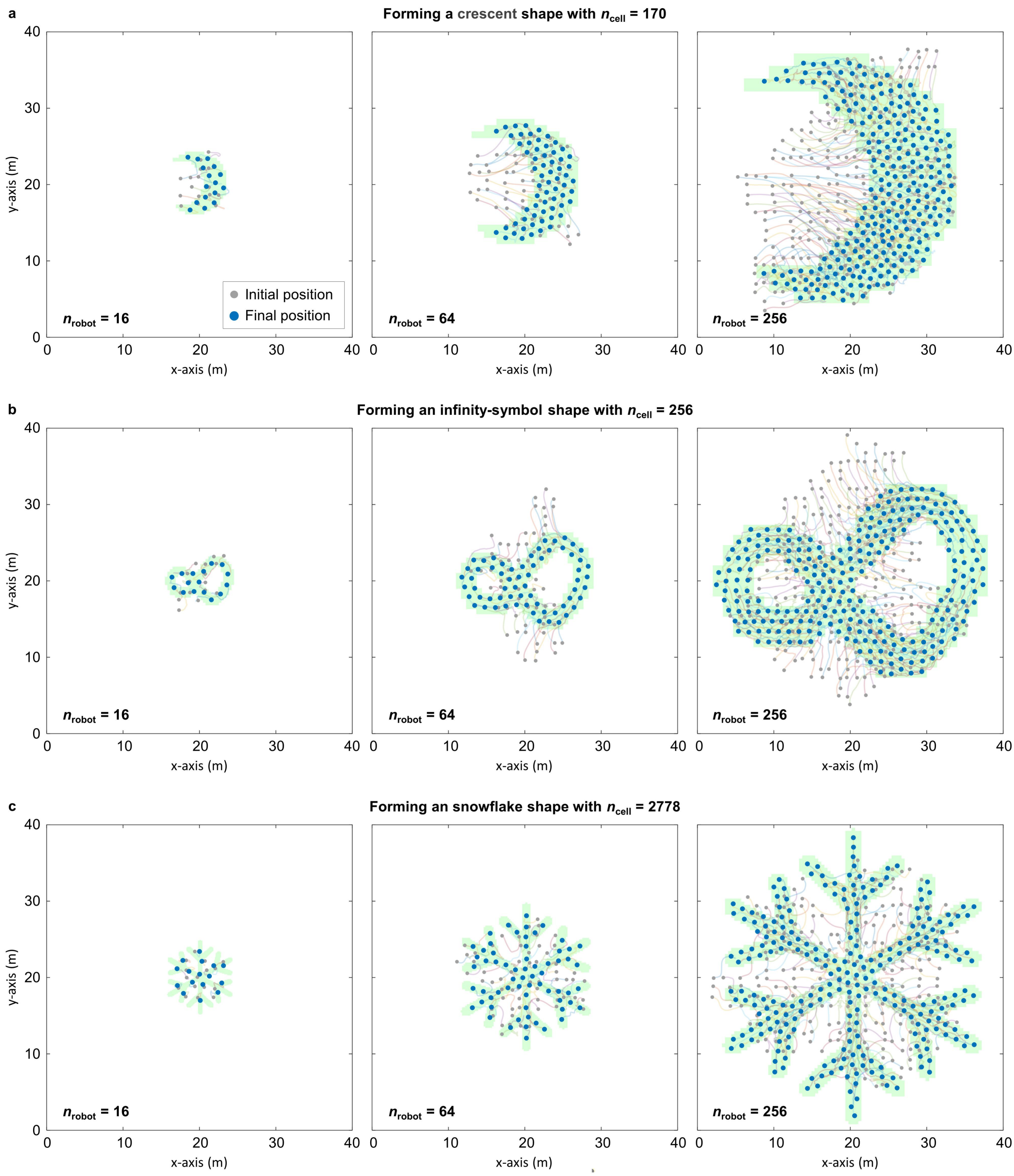

Supplementary Figure 5: Simulation results to demonstrate the adaptability of the proposed method given different desired shapes, n_{cell}, and n_{robot}. **a** Crescent shape assembly. **b** Infinity shape assembly. **c** Snowflake shape assembly.

severely. For example, when $n_{\text{cell}} = 2778 \gg n_{\text{robot}} = 64$ (the second subfigure in Supplementary Figure 5c), the desired shapes can still be well assembled.

4.2.3 Example 3: Maneuverability

This example demonstrates that the proposed method can track moving shapes. In this example, a swarm of 128 robots sequentially assemble five letters, "R", "O", "B", "O", and "T", from random initialization. These five letter shapes are generated by loading predesigned images and sent to all the robots one by one. Moreover, seven out of 128 (about 5%) robots in the swarm are selected informed robots that know the desired sinusoid trajectory of the shape.

As shown in Supplementary Figure 6, the proposed method can assemble the desired shape while steering the shape to move as expected. The distributed negotiation on the desired shape can converge quickly as shown in Supplementary Figure 6a. Supplementary Figure 6c shows the performance metrics in this example. It can be seen that the two metrics can converge to their ideal states promptly

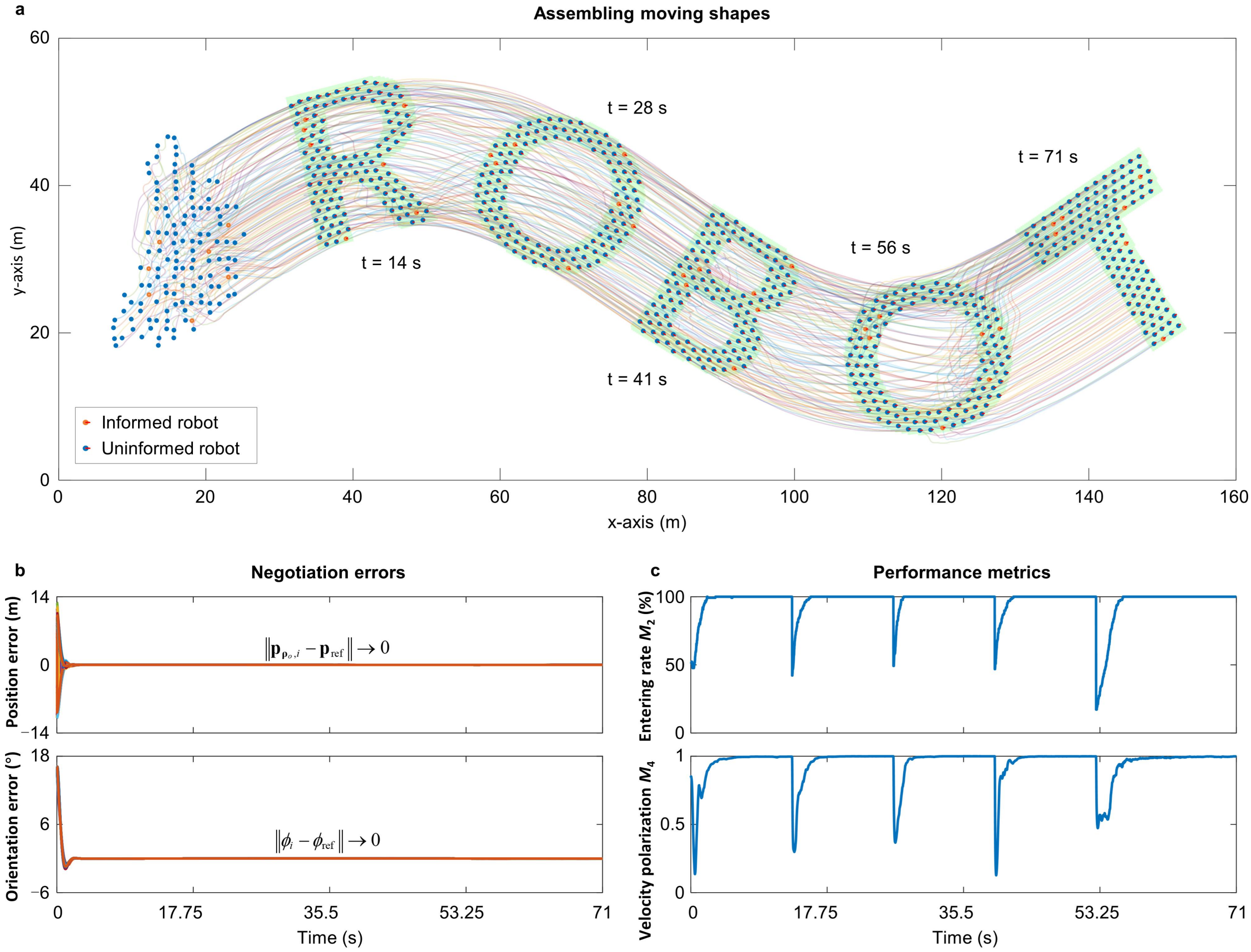

Supplementary Figure 6: Simulation results for illustrating the maneuverability of the proposed method. **a** Swarm trajectory. There are 128 robots. **b** Negotiation errors of the shape translation and orientation with respect to the desired reference. **c** Performance metrics.

after the desired shape is switched.

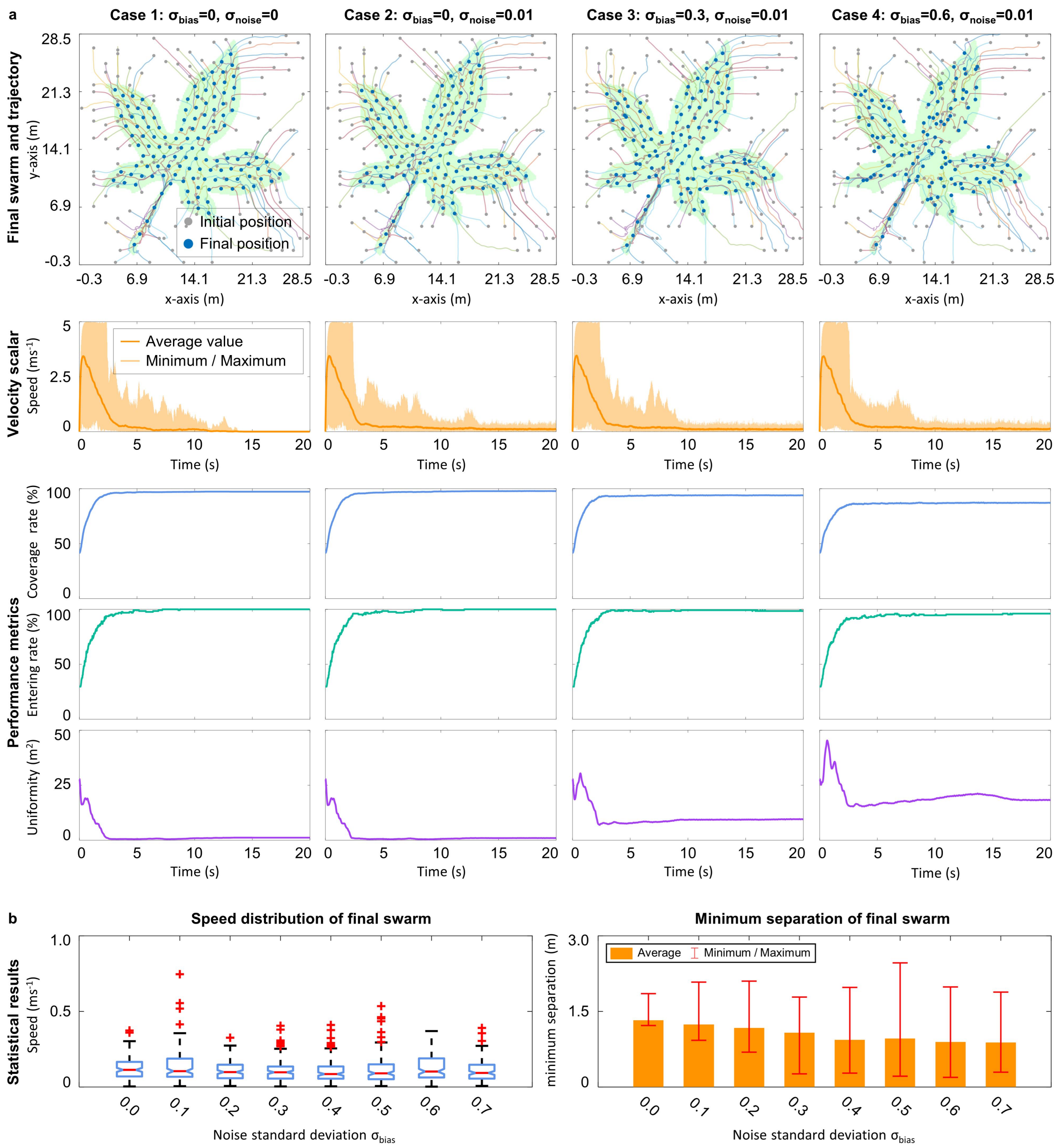

Supplementary Figure 7: The influence of measurement errors. Constant biases and white noises are added to the position and velocity measurements of each robot. The biases for each robot are randomly generated in the beginning and remain the same during the entire process. The white noises are generated at every time step. The impact of the constant biases is that the performance described by the three metrics drops gradually as the magnitude of biases increases. The impact of the white noises is that the robots would constantly adjust their positions slightly due to the time-varying noises.

4.2.4 Example 4: Influence of measurement errors

This example examines the influence of measurement errors on the control performance. In the presence of different levels of measurement biases and white noises, the shape assembly results are shown in Supplementary Figure 7. In general, as the level of measurement errors increases, the performance of the proposed method would degenerate gradually in terms of the three performance metrics. Intuitively, the finally assembled shape would be deformed in the presence of measurement errors, which is in general a common phenomenon for cooperative estimation and control tasks [7,8].

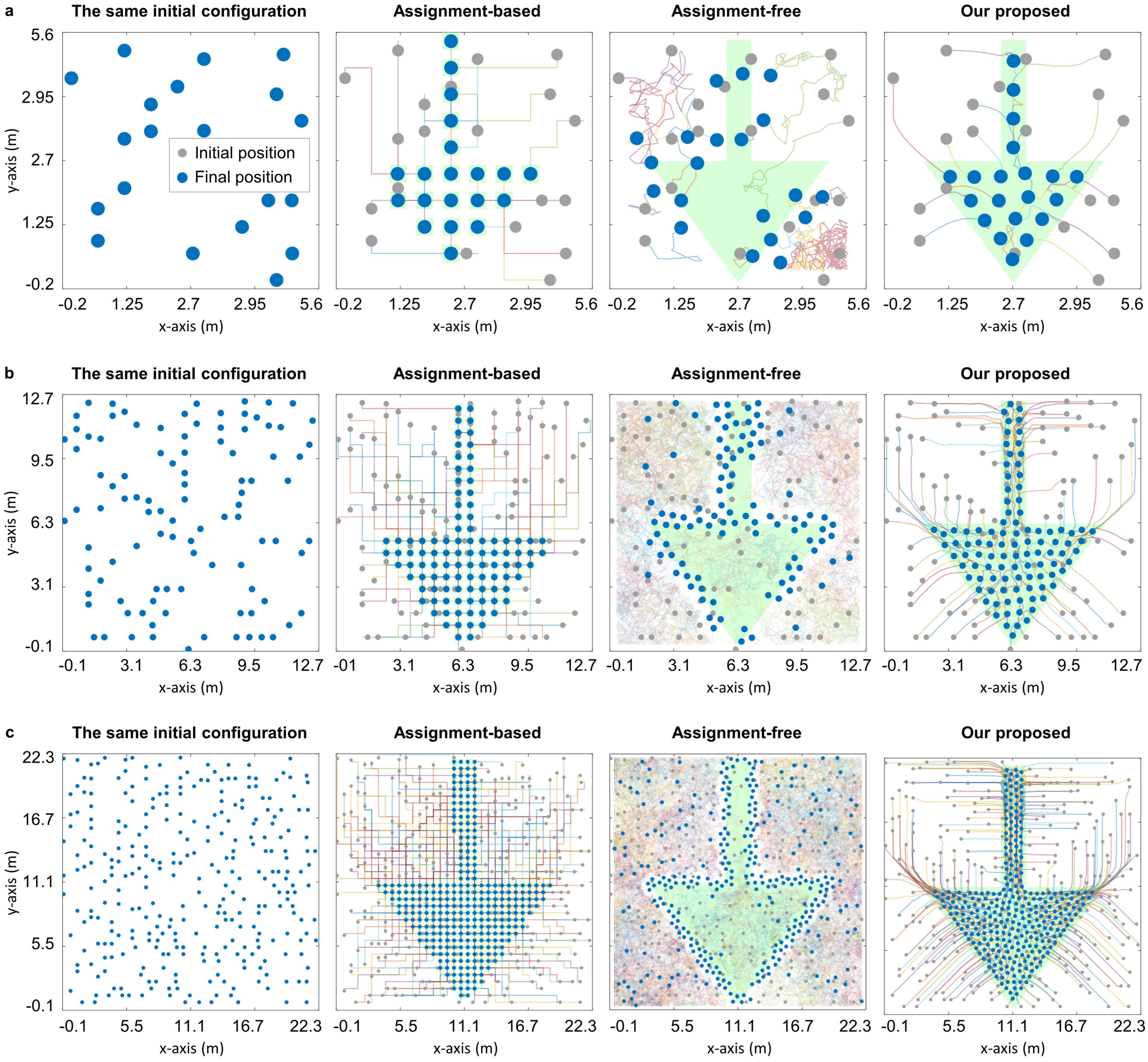

Supplementary Figure 8: Comparison between the proposed method, the assignment-based one in [9] and the assignment-free one in [10]. The desired shape is an arrow. **a** The case of 20 robots. **b** The case of 100 robots. **c** The case of 300 robots.

4.2.5 Example 5: More comparison results

In this example, the proposed method is compared with two state-of-the-art ones in [9] and [10]. The simulation result of assembling an "N" letter shape is shown in Figure 4 in the main text. Here, we present another scenario where different numbers of robots assemble an arrow shape. The simulation results are shown in Supplementary Figure 8. As can be seen, the proposed method leads to smoother and shorter trajectories.

The parameters of the three algorithms are shown in Supplementary Table 2. It is noted that the parameters of *communication period* for the three algorithms have different values. The explanation is given below. The communication period is usually selected to be the same as the *control period*. This is the case for our proposed control strategy where both the control and communication periods equal 10 ms. However, the assignment-based method in [9] requires different values of the two parameters. It also requires another parameter called *plan period*. The relationship between the three parameters must satisfy certain conditions. In particular, denote the communication frequency as f_{comm}. Then, the communication period is equal to $1/f_{\mathrm{comm}}$. Let Δt denote the plan period. Then, the three parameters must satisfy $2/f_{\mathrm{comm}} = \Delta t > l/v_m$ [9], where l is the grid size and v_m is the speed of a robot. Finally, the value of the communication period of the assignment-free method [10] is fine-tuned. The communication period can also be selected as 10 ms, but the performance can be improved when it is selected as 100 ms. That is because too frequent updates of the neighbors' states may downgrade the edge detection performance.

Supplementary Table 2: Simulation parameters of the three methods.

Parameters	Assignment-based	Assignment-free	The proposed
Body size (m)	0.15	0.15	0.15
Sensing range (m)	1.30	1.30	1.30
Avoidance distance (m)	-	0.55	0.55
Grid size (m)	0.60	-	-
Light-detection distance (m)	-	0.70	-
Border size (m)	-	0.00	-
Control period (ms)	10	10	10
Plan period (ms)	600	-	-
Communication period (ms)	300	100	10

5 Experiments on 50 Robots

5.1 Robotic hardware platform

Every robot built for the experiments has a radius of 0.15 m and a height of 0.25 m. It consists of four major modules: 1) a master controller for robot motion control; 2) a wireless transceiver for information transmission; 3) four driving rotors with Mecanum wheels providing omnidirectional maneuverability; and 4) an LED belt exhibiting the robot's moving intention. More specifically, the main controller carries an STM32F4 microprocessor with a 168 Mhz clock and 192 kB random-access memory, and an ICM-20948 motion-tracking device for sensing acceleration and angular velocity with accuracy up to $0.0006\ \mathrm{m/s^2}$ and $0.0153\ ^\circ/\mathrm{s}$. A real-time operating system FreeRTOS is embedded in the microprocessor to handle the robot's basic behaviors, LED belt display, and interact with the workstation. The microprocessor controls the robot's motion at a rate of 100 Hz. Each robot is equipped with a 2.4 GHz Wi-Fi transceiver, which is mainly used to connect to the workstation's wireless router. The robot moves in a two-dimensional plane using four Mecanum wheels driven by DC motors. A dual H-bridge driver controls the speed and direction of each motor independently, allowing for omnidirectional motion. The maximum speed of each wheel is limited to 3.5 m/s.

Each robot can exhibit its motion intention by using the LED belt on the inner edge of the robot's dome. Each LED can show four types of colors: red, green, blue, and white. Red, green, and blue correspond to the magnitudes of $\mathbf{v}_i^{\mathrm{ent}}$, $\mathbf{v}_i^{\mathrm{exp}}$, and $\mathbf{v}_i^{\mathrm{int}}$, respectively. White corresponds to the velocity magnitude of the robot. When the number of LEDs exhibiting a certain color is large, the magnitude of the corresponding quantity is large. In particular, denote n_{led} as the total number of LEDs in the belt, and $\mathbf{v}_{\max} \in \mathbb{R}$ as the maximum speed of the robot. The number of white LEDs is designed as

$$n_{\mathrm{white}} = \lfloor n_{\mathrm{led}}(\mathbf{v}_{\max} - \|\mathbf{v}\|)/\mathbf{v}_{\max} \rfloor$$

where $\lfloor \cdot \rfloor$ means round down. The smaller the velocity command is, the more white LEDs there are. If all LEDs are white, the robot intends to stop moving. Let n_{red}, n_{green}, and n_{blue} refer to the number of red, green, and blue LEDs, respectively. Then, $n_{\mathrm{red}} + n_{\mathrm{green}} + n_{\mathrm{blue}} = n_{\mathrm{led}} - n_{\mathrm{white}}$. We design

$$\begin{aligned}
n_{\mathrm{red}} &= \left\lfloor (n_{\mathrm{led}} - n_{\mathrm{white}}) \frac{\|\mathbf{v}_i^{\mathrm{ent}}\|}{\|\mathbf{v}_i^{\mathrm{ent}}\| + \|\mathbf{v}_i^{\mathrm{exp}}\| + \|\mathbf{v}_i^{\mathrm{int}}\|} \right\rceil, \\
n_{\mathrm{green}} &= \left\lfloor (n_{\mathrm{led}} - n_{\mathrm{white}}) \frac{\|\mathbf{v}_i^{\mathrm{exp}}\|}{\|\mathbf{v}_i^{\mathrm{ent}}\| + \|\mathbf{v}_i^{\mathrm{exp}}\| + \|\mathbf{v}_i^{\mathrm{int}}\|} \right\rceil, \\
n_{\mathrm{blue}} &= \left\lfloor (n_{\mathrm{led}} - n_{\mathrm{white}}) \frac{\|\mathbf{v}_i^{\mathrm{int}}\|}{\|\mathbf{v}_i^{\mathrm{ent}}\| + \|\mathbf{v}_i^{\mathrm{exp}}\| + \|\mathbf{v}_i^{\mathrm{int}}\|} \right\rceil,
\end{aligned}$$

where $\lfloor \cdot \rceil$ is the operation to round to the nearest integer.

5.2 Experimental setup

All experiments are conducted in a rectangular arena with the dimension as $8\ \mathrm{m} \times 7\ \mathrm{m} \times 3\ \mathrm{m}$. There are five key components in the experiments: 1) 50 robot swarm, 2) a workstation, 3) wireless routers for connecting the robot swarm and workstation, 4) a motion capture system for positioning robots, and 5) an ultra-wide angle camera used to film experiments. These components are detailed as follows.

Robot swarm: A swarming system of 50 ground robots was developed. All robots in the swarm are homogeneous and have no unique identities among them. Although different robots may have different IP addresses and position markers, these labels are not used in the algorithms.

Workstation: Limited by the computing power of the robot's microprocessor, a workstation is used to control the robot swarm in a multithreaded manner. The workstation carries a 3.6 GHz and 64 bit six-core Xeon processor, and 32 GB random-access memory. In the experiments, each robot corresponds to an independent control thread in the workstation and communicates with its corresponding thread using wireless routers. In this way, each robot has two controllers. One runs the proposed algorithm in the workstation, and the other is onboard and tracks the control commands sent from the workstation.

Wireless router: To avoid channel congestion and reduce transmission latency, six wireless routers are used to establish the connection between the robots and their thread in the workstation. In the experiments, it is primary to ensure real-time and efficient transmission while a certain amount of packet loss can be tolerated. Thus, the communication along this connection uses standard UDP protocol to complete the information transmission at a rate of $40\ \mathrm{Hz}$.

VICON system: A VICON motion capture system is used to track the robot's motion. All motion states, including position and heading, are acquired by the workstation at an update rate of $100\ \mathrm{Hz}$ via the VICON-DataStream software development kit. Then, the robot's motion velocity and heading rate can be calculated by using the method of forward differencing. In the workstation, there is an additional thread for receiving and calculating all motion states of the robots.

Camera: An ultra-wide angle camera is mounted above the experimental arena to record the experiments at 4 K and 60 fps. The operation and parameter setting of the camera can be done via

cell phone. In some experiments, the brightness and exposure of the camera are adjusted to increase contrast.

5.3 Parameter setting

The parameter settings of all experiments in this paper are shown in Supplementary Table 3. Although the same set of parameters can still effectively work across different experiments, we fine-tune them to gain better performance. The criterion for parameter tuning is explained below. In the shape-assembly experiments, all the parameters are the same except that σ_2 varies mildly in different scenarios. This is because robots may require different exploring strengths for different complex shapes. In the cargo-transportation experiment, σ_1 is an important parameter. Here, it is tuned to be a little large so that robots can enter the moving shape and encircle the cargo as quickly as possible. In the space-exploration experiment, σ_1 and κ_3 are tuned to be a little large so that the robots can flood into the space quickly while avoiding collision.

Supplementary Table 3: Experiment parameter settings

Experiments		Parameters						
		κ_1	σ_1	σ_2	κ_3	α	c_1	c_2
Complex shape assembly	Fig. 3A	2.0	0.5	6.0	10.0	0.8	1.6	1.6
	Fig. 3C	2.0	0.5	5.0	10.0	0.8	1.6	1.6
	Fig. 4	2.0	0.5	6.0	10.0	0.8	1.6	1.6
Cargo transportation	Fig. 5	2.0	2.0	6.0	6.0	0.8	1.6	1.6
Space exploration	Fig. 6A	2.5	2.0	5.0	15.0	0.8	1.6	1.6
	Fig. 6B	2.5	3.0	8.0	15.0	0.8	1.6	1.6

Parameter ℓ_{cell} may influence the smoothness of the control command. When ℓ_{cell} is too big (e.g., equal to the sensing radius of each robot), the number of cells that are inside the sensing radius of each robot would be small. In this case, a cell leaving or entering the sensing radius would constitute a major change that may cause an abrupt change in the control command. By contrast, when ℓ_{cell} is small, the number of cells that are inside the sensing radius of each robot is large. In this case, a cell leaving or entering the sensing radius would cause a smaller change in the control command and hence the smoothness is enhanced.

Selecting an appropriate sampling rate is important to achieve accurate and stable performance. Discretization can affect the precision of a dynamic system. For the negotiation/consensus process, an implementation obtained from discretizing a finite-time-convergent continuous-time algorithm

would converge to a neighborhood of zero (instead of exactly zero) [11]. Similarly, discretization would cause a loss of information which may result in poorer control performance [12, Chapter 14.2]. The digital implementation of our control algorithm uses a zero-order hold. Similar techniques are also used in [9, 13, 14]. Nevertheless, the impact of discretization is neglectable if the sampling rate is properly selected. General guidelines on how to select appropriate sampling rates can be found in [12, Chapter 14.2]. For example, the sampling rate of the consensus process in our simulation and experiments is 100 Hz. The resulting consensus errors of the position and orientation of the desired shape are smaller than the level of 10^{-2} even when the shape tracks a time-varying trajectory. The experimental results confirm a satisfactory level of performance of the system.

6 Convergence Analysis

We next present the convergence analysis of the proposed negotiation and control algorithms. The output of the consensus process is used by the control process, but the negotiation process does not rely on the control process. This relationship is illustrated by Supplementary Figure 9.

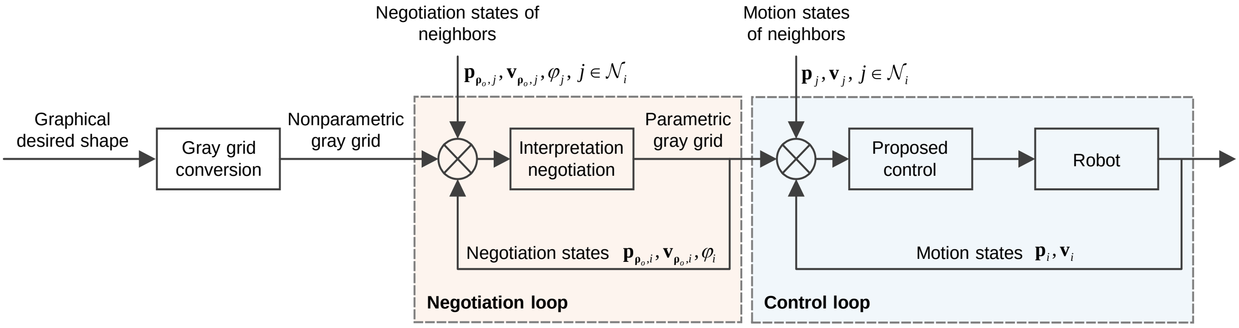

Supplementary Figure 9: The structure of the proposed method.

It is notable that the consensus process converges much faster than the control process. The fundamental reason is that the consensus process is a numerical computational process that involves only virtual states (i.e., the position and orientation of the desired shape). These states are *not* subject to physical dynamics. By contrast, the control process is subject to real physical dynamics and constraints such as the dynamics of the robots and the saturation of the velocity and acceleration. Therefore, the control process evolves much slower. The experimental and simulation results support this observation. For instance, it can be seen from Figure 3c in the main text that the consensus process converges more than 5 times faster than the control process (the convergence time of the consensus process is about 3 seconds whereas that of the control process is about 20 seconds).

Since the consensus process converges much faster, it can be viewed as a fast inner loop while the control process is a slow outer loop. Then, we can analyze the two processes separately. That is,

when we analyze the convergence of the control process, we can assume that the consensus process has already converged.

6.1 Convergence Analysis of Negotiation Algorithms

6.1.1 Preliminaries

We first introduce some preliminary results.

Lemma 1 ([15]). *Suppose that function $V(t) : [0, \infty) \to [0, \infty)$ is differentiable and satisfies*

$$\dot{V}(t) \le -cV(t)^{\alpha}$$

where $c > 0$ and $0 < \alpha < 1$ are two constants. Then, $V(t)$ will reach zero at finite time $t^* \le V(0)^{1-\alpha}/[c(1-\alpha)]$ and $V(t) = 0$ for all $t \ge t^*$.

Lemma 2 ([16]). *Let $z_1, z_2, ..., z_m \ge 0$ and $0 < \beta \le 1$. Then*

$$\left(\sum_{i=1}^{m} z_i\right)^{\beta} \le \sum_{i=1}^{m} z_i^{\beta}.$$

Lemma 3 ([17]). *Suppose function g satisfies $g(z_i, z_j) = -g(z_j, z_i), i, j \in \{1, 2, ..., m\}, i \ne j$. Then, for a set of numbers $y_1, y_2, ..., y_m$, it holds that*

$$\sum_{i=1}^{m}\sum_{j=1}^{m} y_i g(z_i, z_j) = \frac{1}{2}\sum_{i=1}^{m}\sum_{j=1}^{m}(y_i - y_j)g(z_i, z_j).$$

6.1.2 Convergence analysis

We next analyze the convergence of the negotiation algorithm in (3) and (4) in the main text. We only consider the case where there are no informed robots. In particular, the negotiation algorithm in (3) in the main text can be written as

$$\mathbf{v}_{\rho_o,i} = -\frac{c_1}{|\mathcal{N}_i|}\sum_{j=1}^{n_{\text{robot}}} a_{ij}\text{sig}\left(\mathbf{p}_{\rho_o,i} - \mathbf{p}_{\rho_o,j}\right)^{\alpha} + \frac{1}{|\mathcal{N}_i|}\sum_{j=1}^{n_{\text{robot}}} a_{ij}\mathbf{v}_{\rho_o,j} \tag{4}$$

where $\text{sig}(\cdot)^\alpha = \text{sign}(\cdot)|\cdot|^\alpha$, and $a_{ij} \in \{0, 1\}$. If $(i, j) \in \mathcal{E}$, then $a_{ij} = 1$, otherwise $a_{ij} = 0$.

Lemma 4 (Properties of sig function)**.** *For any vector* $\mathbf{z} = [z_1, ..., z_m]^{\mathrm{T}}$*, it holds that* $\text{sig}(-\mathbf{z})^\alpha = -\text{sig}(\mathbf{z})^\alpha$ *and* $\mathbf{z}^{\mathrm{T}}\text{sig}(-\mathbf{z})^\alpha \geq \|\mathbf{z}\|^{\alpha+1}$*, where* $\|\cdot\|$ *is the Euclidean norm.*

Proof. First, $\text{sig}(-\mathbf{z})^\alpha = \text{sign}(-\mathbf{z})|-\mathbf{z}|^\alpha = -\text{sign}(\mathbf{z})|\mathbf{z}|^\alpha = -\text{sig}(\mathbf{z})^\alpha$. Second, $\mathbf{z}^{\mathrm{T}}\text{sig}(-\mathbf{z})^\alpha = \sum_{i=1}^m z_i \text{sign}(z_i)|z_i|^\alpha = \sum_{i=1}^m |z_i|^{\alpha+1} = \sum_{i=1}^m (|z_i|^2)^{\frac{\alpha+1}{2}}$. Based on Lemma 2, we have $\mathbf{z}^{\mathrm{T}}\text{sig}(-\mathbf{z})^\alpha = \sum_{i=1}^m (|z_i|^2)^{\frac{\alpha+1}{2}} \geq (\sum_{i=1}^m |z_i|^2)^{\frac{\alpha+1}{2}} = \|\mathbf{z}\|^{\alpha+1}$. □

With the above preparation we are ready to present the following convergence result. It should be noted that the convergence relies on an assumption of the underlying graph $\mathcal{G}$. In particular, $\mathcal{G}$ is assumed to be connected and fixed. This assumption is valid when the initial graph is connected and the consensus process converges much faster than the control process. In particular, we can suppose that, when the consensus process converges, the swarm configuration is almost unchanged and hence the graph remains fixed and connected. In fact, a simple method that can strictly ensure the assumption of the underlying graph would be to run an initialization process, during which we wait for the consensus process to converge first. We did not do this because the present strategy already works effectively.

The design of the consensus algorithm in (4) is inspired by the work in [16] and our previous work in [3]. The proof of the finite-time convergence given below is inspired by the method in [16].

Theorem 1 (Convergence of negotiation)**.** *If* $\mathcal{G}$ *is undirected, connected, and fixed, under the negotiation algorithm (3) and (4), there exists a finite time* $t^* > 0$ *such that* $\mathbf{p}_{\rho_o,i}(t) = \mathbf{p}_{\rho_o,j}(t)$ *and* $\phi_i(t) = \phi_j(t)$ *for all* $t \geq t^*$ *and all* $i, j \in \mathcal{V}$.

Proof. Reorganizing (4) and multiplying $|\mathcal{N}_i|$ on both sides gives

$$\sum_{j=1}^{n_{\text{robot}}} a_{ij}\left(\mathbf{v}_{\rho_o,i} - \mathbf{v}_{\rho_o,j}\right) = -c_1 \sum_{j=1}^{n_{\text{robot}}} a_{ij}\text{sig}\left(\mathbf{p}_{\rho_o,i} - \mathbf{p}_{\rho_o,j}\right)^\alpha.$$

Multiplying $\sum_i \mathbf{p}_{\rho_o,i}^{\mathrm{T}}$ on both sides of the above equation yields

$$\sum_{i,j=1}^{n_{\text{robot}}} a_{ij}\mathbf{p}_{\rho_o,i}^{\mathrm{T}}(\mathbf{v}_{\rho_o,i} - \mathbf{v}_{\rho_o,j}) = -c_1 \sum_{i,j=1}^{n_{\text{robot}}} a_{ij}\mathbf{p}_{\rho_o,i}^{\mathrm{T}}\text{sig}(\mathbf{p}_{\rho_o,i} - \mathbf{p}_{\rho_o,j})^\alpha.$$

By applying Lemma 3 and the fact that $a_{ij} = a_{ji}$, we have

$$\sum_{i,j=1}^{n_{\text{robot}}} a_{ij} \left(\mathbf{p}_{\rho_o,i} - \mathbf{p}_{\rho_o,j}\right)^{\mathrm{T}} \left(\mathbf{v}_{\rho_o,i} - \mathbf{v}_{\rho_o,j}\right) = -c_1 \sum_{j=1}^{n_{\text{robot}}} a_{ij} \left(\mathbf{p}_{\rho_o,i} - \mathbf{p}_{\rho_o,j}\right)^{\mathrm{T}} \operatorname{sig}\left(\mathbf{p}_{\rho_o,i} - \mathbf{p}_{\rho_o,j}\right)^{\alpha}. \quad (5)$$

Choose the Lyapunov candidate as

$$V(t) = \frac{1}{2} \sum_{i,j=1}^{n_{\text{robot}}} a_{ij} \left(\mathbf{p}_{\rho_o,i} - \mathbf{p}_{\rho_o,j}\right)^{\mathrm{T}} \left(\mathbf{p}_{\rho_o,i} - \mathbf{p}_{\rho_o,j}\right).$$

Substituting (5) into the time derivative of $V(t)$ yields

$$\dot{V}(t) = \sum_{i,j=1}^{n_{\text{robot}}} a_{ij} \left(\mathbf{p}_{\rho_o,i} - \mathbf{p}_{\rho_o,j}\right)^{\mathrm{T}} \left(\mathbf{v}_{\rho_o,i} - \mathbf{v}_{\rho_o,j}\right) = -c_1 \sum_{j=1}^{n_{\text{robot}}} a_{ij} \left(\mathbf{p}_{\rho_o,i} - \mathbf{p}_{\rho_o,j}\right)^{\mathrm{T}} \operatorname{sig}\left(\mathbf{p}_{\rho_o,i} - \mathbf{p}_{\rho_o,j}\right)^{\alpha}.$$

It then follows from Lemma 4 that

$$\dot{V}(t) \leq -c_1 \sum_{i,j=1}^{n_{\text{robot}}} a_{ij} \left\|\mathbf{p}_{\rho_o,i} - \mathbf{p}_{\rho_o,j}\right\|^{\alpha+1} = -c_1 \sum_{i,j=1}^{n_{\text{robot}}} \left(a_{ij}^{\frac{2}{\alpha+1}} \left\|\mathbf{p}_{\rho_o,i} - \mathbf{p}_{\rho_o,j}\right\|^2\right)^{\frac{\alpha+1}{2}}.$$

Since $a_{ij} \in \{0, 1\}$, we have $a_{ij}{}^{\frac{2}{\alpha+1}} = a_{ij}$. It then follows from Lemma 2 that

$$\dot{V}(t) \leq -c_1 \left(\sum_{i,j=1}^{n_{\text{robot}}} a_{ij} \left\|\mathbf{p}_{\rho_o,i} - \mathbf{p}_{\rho_o,j}\right\|^2\right)^{\frac{\alpha+1}{2}} = -c_1 \left[2V(t)\right]^{\frac{\alpha+1}{2}} = -2^{\frac{\alpha+1}{2}} c_1 V(t)^{\frac{\alpha+1}{2}}$$

Thus, by Lemma 1, there exist $c = 2^{\frac{\alpha+1}{2}} c_1$ and $t_1^* \leq V(0)/[c(1-\alpha)]$ such that $V(t) = 0$ for all $t \geq t_1^*$.

The convergence proof of algorithm (4) in the main text is similar to algorithm (3). We can also conclude that there exists at finite time t_2^* such that all robots' negotiations on shape orientation will reach an agreement before t_2^*. Let $t^* = \max\{t_1^*, t_2^*\}$. Then, both $\mathbf{p}_{\rho_o,i}(t) = \mathbf{p}_{\rho_o,j}(t)$ and $\phi_i = \phi_j$ for all $t \geq t^*$ and all $i, j \in \mathcal{V}$. □

6.2 Convergence Analysis of Control Algorithms

6.2.1 Preliminaries

We need to introduce a necessary preliminary result.

Theorem 2 (Direction of mean-shift [18]). *Let* $\mathbf{x}_i \in \mathbb{R}^2$ *be a data point and* $D = \{\mathbf{x}_i\}$ *be a set of*

finite data points. Suppose $g(\cdot)$ is a kernel function. The mean-shift vector defined for any $\mathbf{y} \in \mathbb{R}^2$,

$$\mathbf{m}(\mathbf{y}) = \frac{\sum_{\mathbf{x}_i \in D} g\left(\|\mathbf{y} - \mathbf{x}_i\|\right) \mathbf{x}_i}{\sum_{\mathbf{x}_i \in D} g\left(\|\mathbf{y} - \mathbf{x}_i\|\right)} - \mathbf{y}, \tag{6}$$

points toward the direction of the maximum increase of the data point density, that is the gradient direction.

The above theorem indicates a well-known property of the classic mean-shift algorithm for density maximization. Since the proposed shape-exploration command $\mathbf{v}_i^{\exp}$ in (6) in the main text is an adapted mean-shift algorithm, Theorem 2 will be useful.

6.2.2 Objective function and assumptions

Consider the objective function $J(t)$ defined as

$$J(t) = \sum_{i=1}^{n_{\text{robot}}} J_i(t) = \sum_{i=1}^{n_{\text{robot}}} \left\lfloor h\, \xi_{\rho_i,\text{c}}(t) \right\rceil \tag{7}$$

where h is the number of gray levels, $\xi_{\rho_i,\text{c}}$ is the gray value of the cell occupied by robot i in $\mathcal{F}_{\text{gray,c}}$, and $\lfloor \cdot \rceil$ is rounding to the nearest integer. Here, h is a constant and hence can be removed from the objective function. However, we retain it because $0 \le \xi_{\rho_i,\text{c}}(t) \le 1$ but $h\xi_{\rho_i,\text{c}}(t)$ is an integer so that we can apply Theorem 3. Note that $J_i(t) = \lfloor h\, \xi_{\rho_i,\text{c}}(t) \rceil$ is the value of robot $i = 1, \ldots, n_{\text{robot}}$.

Since $J(t) = 0$ if and only if all robots enter $\mathcal{F}_\text{c}$, we can prove the convergence of the control dynamics by showing that $J(t)$ converges to zero. To that end, we introduce the following theorem from [9].

Theorem 3 (Almost sure convergence [9])**.** *Let $P\{X\}$ denote the probability of event X occurring. For any function $J(t) \in \mathbb{Z}^{\ge 0}$, it will almost surely converge to zero if:*

1) $J(t)$ is always bounded from above: $\exists a \in \mathbb{Z}^{\ge 0}$, s.t. $\forall t, J(t) \le a$;

2) $J(t)$ is always non-increasing: $\forall t_2 \ge t_1, J(t_2) \le J(t_1)$;

3) $J(t)$ is strictly decreasing with nonzero probability: $\exists \tau, \epsilon > 0$, s.t. $\forall t, P\{J(t+\tau) \le J(t) - 1 | J(t) \ne 0\} \ge \epsilon$.

To apply this theorem, considering the high complexity of the overall system, we introduce the following mild assumptions.

1) Convex desired shape. Although the proposed control method works effectively for non-convex shapes, we only consider convex shapes in the convergence analysis. In particular, the shape should

be such that, for a robot located in any position, the black cells and the non-black cells inside the robot's sensing radius form two convex sets, respectively. In this way, the convergence proof will be simplified for the robots that are near the boundary of the desired shape.

2) Large desired shape. We assume that the desired shape is sufficiently large to accommodate all n_{robot} robots. If this assumption is not satisfied, it is possible that the robots may not be able to move into the desired shape and $J(t)$ may not reach zero. For example, suppose each robot occupies a space of πr_{avoid}^2 and the space of the desired shape should be at least $n_{\text{robot}}\pi r_{\text{avoid}}^2$.

3) Stationary desired shape. Although the proposed control method can handle moving desired shapes, we only consider the case of stationary desired shapes. That is $v_{\text{ref}} = 0$. Moreover, since the velocity alignment term in (7) is designed for tracking moving desired shapes, it is ignored in the convergence proof as well. Experimental results verify that the absence of this term does not affect the convergence of the system for stationary desired shapes. In this way, the control command (7) becomes a pure repelling velocity command for inter-robot collision avoidance.

4) Sparse initial configuration. We assume that in the initial configuration the robots are located sparsely so that the distance between any two robots is greater than r_{avoid}. Without this assumption, robots may have large repelling velocities initially so that $J(t)$ may increase.

6.2.3 Convergence of the objective function

With the above preparation, we are ready to analyze the system convergence. We will prove that the three conditions in Theorem 3 are all valid one by one. The intuition is illustrated by the simulation example in Supplementary Figure 10, which shows that $J_i(t)$ for all i and $J(t)$ are decreasing.

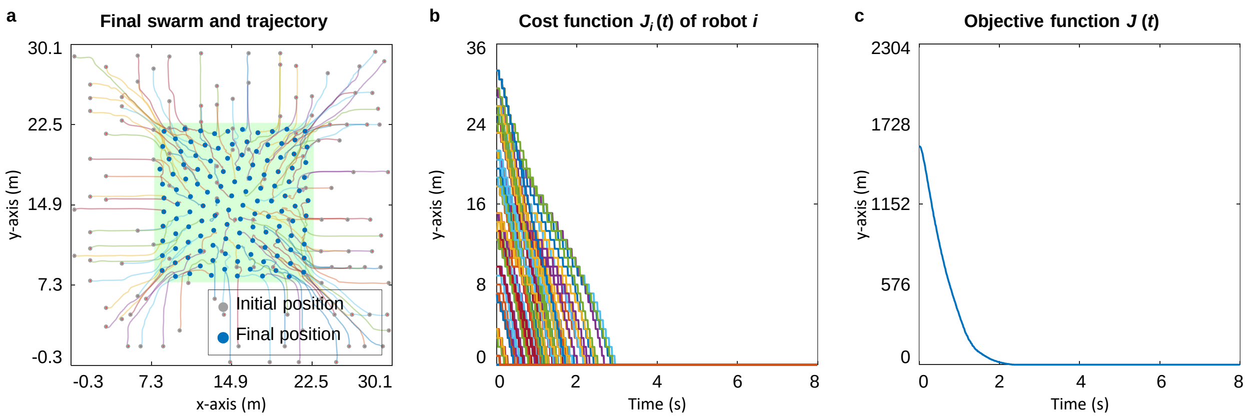

Supplementary Figure 10: An example to illustrate the mathematical property that $J(t)$ and $J_i(t)$ decrease zero.

First, we show that the first condition in Theorem 3 is valid.

Lemma 5 (Boundedness of $J(t)$)**.** *The objective function $J(t)$ is bounded from above for all $t = 0, 1, 2, \ldots$ and specifically satisfies $0 \leq J(t) \leq nh$.*

Proof. It follows from (1) in the main text that $\xi_{\boldsymbol{\rho}_i,\mathrm{c}}$ takes a value in $[0, 1]$: if $\boldsymbol{\rho}_i$ is black, then $\xi_{\boldsymbol{\rho}_i,\mathrm{c}} = 0$; if $\boldsymbol{\rho}_i$ is white, then $\xi_{\boldsymbol{\rho}_i,\mathrm{c}} = 1$; otherwise, $\boldsymbol{\rho}_i$ is gray and $\xi_{\boldsymbol{\rho}_i,\mathrm{c}} \in (0, 1)$. Therefore, $\lfloor h\, \xi_{\boldsymbol{\rho}_i,\mathrm{c}}(t) \rceil$ is an integer in $[0, h]$ and hence $J(t) = \sum_{i=1}^{n_{\mathrm{robot}}} \lfloor h\, \xi_{\boldsymbol{\rho}_i,\mathrm{c}}(t) \rceil$ is an integer in $[0, nh]$. □

Second, we show that the second condition in Theorem 3 is valid. This is to show that $J(t)$ is always non-increasing.

Lemma 6 (Non-increasing of $J(t)$)**.** *The objective function $J(t)$ is non-increasing for $t = 0, 1, 2, \ldots$.*

Proof. The idea of the proof is to show that $J_i(t)$ is non-increasing for any $i \in \{1, \ldots, n_{\mathrm{robot}}\}$ and hence $J(t) = \sum_{i=1}^{n_{\mathrm{robot}}} J_i(t)$ is non-increasing. This idea is illustrated by Supplementary Figure 10.

We consider the following two scenarios.

- Scenario 1: robot i is inside the desired shape (see Supplementary Figure 11a).

When robot i is inside the desired shape, it is driven jointly by $\mathbf{v}_i^{\exp}$ and $\mathbf{v}_i^{\mathrm{int}}$. We further examine two cases.

Case 1.1: robot i is inside the desired shape but far away from the shape boundary (see the left subfigure of Supplementary Figure 11a). In this case, since the maximum velocity of each robot is limited and the sampling time is sufficiently small, it is impossible for robot i to move out of the shape at the next time step $t + 1$. As a result, $J_i(t)$ would not increase. Moreover, on the one hand, $\mathbf{v}_i^{\exp}$ points to the highest density point of the unoccupied black cells due to Theorem 2. On the other hand, if there are robots inside the collision range of robot i, then $\mathbf{v}_i^{\mathrm{int}}$ points from the highest density of the surrounding robots. As a result, the overall velocity would drive robot i away from the surrounding robots to the highest density of surrounding black cells.

Case 1.2: robot i is inside the desired shape but sufficiently close to the boundary so that the robot may possibly move out of the shape in the next time step (see the right subfigure of Supplementary Figure 11a). In this case, $\mathbf{v}_i^{\exp}$ points to the inside of the desired shape (i.e., the highest density point of black cells). We next show that the overall velocity is impossible to point to the outside of the shape so that robot i moves out of the shape. The proof is by contradiction. Suppose that the overall velocity of robot i points to the outside of the shape. Since $\mathbf{v}_i^{\exp}$ points to the inside, we know that $\mathbf{v}_i^{\mathrm{int}}$ must point to the outside and its magnitude must be greater than $\mathbf{v}_i^{\exp}$. It indicates that robot i must be sufficiently close to one or multiple inner robots so that the repelling velocity generated by the inner robots is great. The sufficient closeness can be generated due to two possibilities.

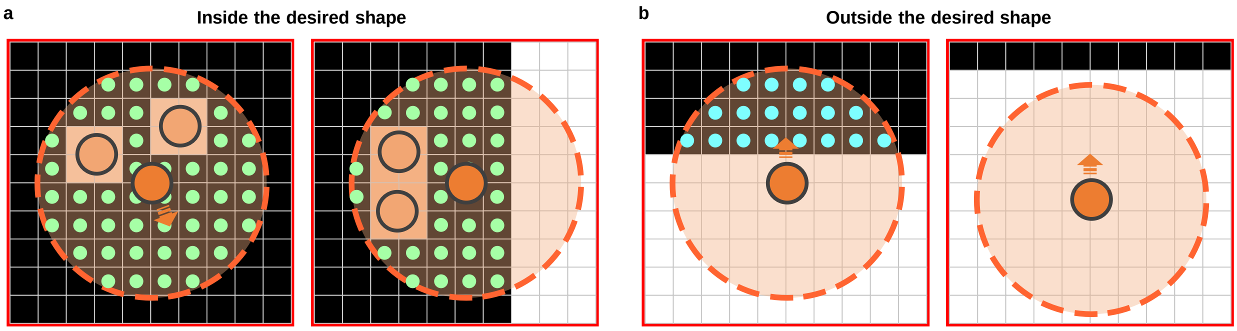

Supplementary Figure 11: An illustration of cases in the proof of Lemma 6. **a** Robot i is inside the desired shape. **b** Robot i is outside the desired shape.

The first possibility is that robot i approaches the inner robots actively to reach a sufficiently small separation. It is however impossible for the repelling velocity to be greater than the exploration velocity since robot i would already stop approaching the inner ones when the repelling velocity increases to certain values. The second possibility is that the inner ones approach robot i actively to reach a sufficiently small separation. This is also invalid. On the one hand, the inner robots would not actively approach a robot sufficiently close based on the analysis in Case 1.1. On the other hand, when the inner robots are close to the shape boundary, their $\mathbf{v}_i^{\mathrm{exp}}$ command would also turn to point to the inside of the shape. Therefore, the overall velocity of a robot would not point to the outside. Therefore, a robot that has entered a shape would not leave and hence $J_i(t)$ does not increase.

- Scenario 2: robot i is located outside of the desired shape (see Supplementary Figure 11b).

This scenario can be further divided into two cases.

Case 2.1: robot i is outside but close to the boundary of the shape so that robot i is surrounded by both black and non-black cells (see the left subfigure of Supplementary Figure 11b). In this case, robot i would be driven by $\mathbf{v}_i^{\mathrm{ent}}$, $\mathbf{v}_i^{\mathrm{exp}}$, and $\mathbf{v}_i^{\mathrm{int}}$. Here, $\mathbf{v}_i^{\mathrm{ent}}$ and $\mathbf{v}_i^{\mathrm{exp}}$ both point to the inner of the shape. Moreover, since the inner agents would not leave the shape as analyzed in Scenario 1, we know that the overall velocity of robot i would not point to the outer of the shape. The detailed analysis is similar to Case 1.2. Therefore, $J_i(t)$ would not increase.

Case 2.2: robot i is outside and far away from the boundary so that the surrounding cells are all non-black (see the right subfigure of Supplementary Figure 11b). In this case, robot i is driven by $\mathbf{v}_i^{\mathrm{ent}}$ and $\mathbf{v}_i^{\mathrm{int}}$. Here, $\mathbf{v}_i^{\mathrm{ent}}$ points to $\boldsymbol{\rho}_{\mathrm{T},i}$, which is the closest and darkest cell. Regarding $\mathbf{v}_i^{\mathrm{int}}$, we know the robots that are close to the shape would not diverge from it based on the analysis in Case 2.1. As a result, they would not attempt to approach robot i to repel it away from the shape. The detailed analysis is similar to Case 1.2. As a result, $J_i(t)$ would not increase. □

Although Lemma 6 shows that $J(t)$ would not increase, it does not indicate whether $J(t)$ will

decrease to zero. We still need to show that the swarm would not get trapped so that $J(t)$ remains the same nonzero value forever. To that end, we introduce the following concept first.

Definition 1 (Deadlock)**.** *A swarm of robots is deadlocked if they all stop moving while they have not entered the desired shape completely.*

We next show that the third condition in Theorem 3 is valid.

Lemma 7 (Decreasing of $J(t)$)**.** *For any t, if $J(t) \neq 0$, then there exists a finite time interval $[t, t+T]$ where the objective function $J(t)$ would strictly decrease by at least one.*

Proof. We prove this by contradiction. Suppose that not all robots have entered the desired shape, but the swarm is deadlocked so that all robots stop moving.

There are two scenarios (see an illustration in Supplementary Figure 12).

Scenario 1: all the robots are deadlocked outside of the desired shape (see Supplementary Figure 12a). This is impossible because if no robots are inside the shape, the outer ones that are close to the shape would move into the inside driven by the $\mathbf{v}_i^{\text{ent}}$ command since there is no repelling velocity that prevents a robot from doing that.

Scenario 2: some robots are inside the shape, and some are outside (see Supplementary Figure 12b). We first examine the robots inside the shape.

Suppose that m robots have already entered the shape, and $m < n_{\text{robot}}$. Since it is assumed that the swarm is deadlocked, the outside robots have stopped moving. Then, the inside robots would disperse in the shape and avoid generating repelling forces among themselves. That is because the inter-robot repulsive command $\mathbf{v}_i^{\text{int}}$ and exploration command $\mathbf{v}_i^{\text{exp}}$ would encourage the robots to disperse. Since the desired shape is assumed to be sufficiently large, the inter-robot distances among the m robots would be greater or close to r_{avoid} so that the repulsive velocity commands among adjacent neighbors would be sufficiently small. In particular, when the desired shape is sufficiently large, the inter-robot repulsive velocity commands would be zero.

Then, consider an outer robot that is outside but about to enter the boundary of the shape. The outer robot would actively approach the inner ones due to the $\mathbf{v}_i^{\text{ent}}$ command. If the control gain κ_3 in $\mathbf{v}_i^{\text{ent}}$ is sufficiently large, the inner robots would be pushed away since the inter-robot repelling command is sufficiently small as analyzed above. Therefore, the outer robot would enter the shape eventually. In summary, when $m < n_{\text{robot}}$ robots are inside the robots, the inner robots disperse so that inter-robot repelling velocity commands are close to zero and hence allow another outside robot to enter. This conflicts with the hypothesis that the outside robots are deadlocked forever.

Therefore, although some individual robots may get trapped outside when "queuing" for entering the shape, the swarm would not get deadlocked forever as analyzed above. As a result, the objective

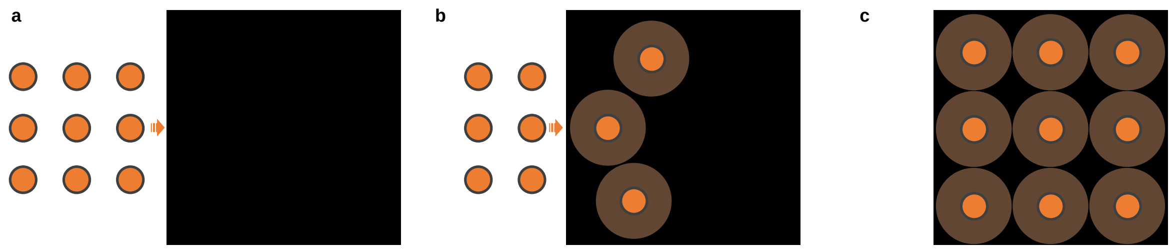

Supplementary Figure 12: An illustration of the deadlocked scenarios in the proof of Lemma 7.

function $J(t)$ would always decrease within a finite time. Once an outer robot enters the shape, $J(t)$ would decrease by at least one. □

Theorem 4 (Convergence of $J(t)$)**.** *The objective function $J(t)$ would eventually converge to zero and hence all the robots would enter the desired shape.*

Proof. Under the assumptions given at the beginning of Section 6.2.3, we can obtain Lemma 5 and Lemma 6, which indicate that $J(t)$ is always bounded from above and $J(t)$ is always non-increasing. Lemma 7 shows that $J(t)$ is strictly decreasing with a probability of one within a finite time. This is a stronger condition than the third condition of Theorem 3, which requires only a nonzero probability. As a result, the third condition of Theorem 3 is satisfied. Therefore, the three conditions in Theorem 3 are satisfied and the convergence of $J(t)$ follows. □

Supplementary References

[1] Ning, B., Han, Q.L. & Lu, Q. Fixed-time leader-following consensus for multiple wheeled mobile robots. *IEEE Trans. Cybern.* **50**, 4381–4392 (2020).

[2] Dong, S., Ren, W. & Wu, Z.G. Observer-based distributed mean-square consensus design for leader-following multiagent Markov jump systems. *IEEE Trans. Cybern.* **51**, 3054–3061 (2021).

[3] Zhao, S. Affine formation maneuver control of multiagent systems. *IEEE Trans. Autom. Control* **63**, 4140–4155 (2018).

[4] Chen, F. & Dimarogonas, D.V. Leaderfollower formation control with prescribed performance guarantees. *IEEE Trans. Control Netw. Syst.* **8**, 450–461 (2021).

[5] Zeng, C., Jian, Y., Vosoughi, S., Zeng, C. & Zhao, Y. Evaluating native-like structures of rna-protein complexes through the deep learning method. *Nat. Commun.* **14**, 1060 (2023).

[6] Groot, J.D. The libraries of the future will be made of DNA. https://theconversation.com/the-libraries-of-the-future-will-be-made-of-dna-86274 (2018).

[7] Cai, Y. & Shen, Y. An integrated localization and control framework for multi-agent formation. *IEEE Trans. Signal Process.* **67**, 1941–1956 (2019).

[8] Zhao, S. & Zelazo, D. Localizability and distributed protocols for bearing-based network localization in arbitrary dimensions. Automatica **698**, 334–341 (2016).

[9] Wang, H. & Rubenstein, M. Shape formation in homogeneous swarms using local task swapping. *IEEE Trans. Robot.* **36**, 597–612 (2020).

[10] Alhafnawi, M., Hauert, S. & ODowd, P. Self-organised saliency detection and representation in robot swarms. *IEEE Robot. Autom. Lett.* **6**, 1487–1494 (2021).

[11] Ceragioli, F., Persis, C.D. & Frasca, P. Discontinuities and hysteresis in quantized average consensus. *Automatica* **47**, 1916–1928 (2011).

[12] Middleton, R.H. & Goodwin, G.C. in *Digital control and estimation: A unified approach.* (Prentice Hall, New Jersey, 1990).

[13] Vsrhelyi, G., Virgh, C., Somorjai, G., Nepusz, T., Eiben, A.E. & Vicsek, T. Optimized flocking of autonomous drones in confined environments. *Sci. Robot.* **3**, eaat3536 (2018).

[14] Li, J., Ning, Z., Hei, S., Leei, C. & Zhao, S. Three-dimensional bearing-only target following via observability-enhanced helical guidance. *IEEE Trans. Robot.* **39**, 1509–1526 (2023).

[15] Bhat, S.P. & Bernstein, D.S. Finite-time stability of continuous autonomous systems. *SIAM J. Control Optim.* **38**, 751–766 (2000).

[16] Wang, L. & Xiao, F. Finite-time consensus problems for networks of dynamic agents. *IEEE Trans. Autom. Control* **55**, 950–955 (2010).

[17] Sun, Y., Feng, L., Li, W. & Shi, H. Finite-time flocking of cucker-smale systems. in *34th Chinese Control Conf.*, pp. 7016–7020 (Hangzhou, China, IEEE, 2015).

[18] Fukunaga, K. & Hostetler, L. The estimation of the gradient of a density function, with applications in pattern recognition. *IEEE Trans. Inf. Theory* **21**, 32–40 (1975).
