# Supplementary material for: Mean-shift exploration in shape assembly of robot swarms
Source: Nat Commun. 2023 Jun 13;14:3476. doi: 10.1038/s41467-023-39251-5 (PMC10264375; doi:10.1038/s41467-023-39251-5)
Supplement: Supplementary file 2 — Description to Additional Supplementary Information [file 41467_2023_39251_MOESM2_ESM.pdf]

Description of Additional Supplementary Files

Supplementary Movie 1: Complex shape assembly - Snowflake shape assembly

Supplementary Movie 2: Complex shape assembly - Letters “ROBOT” assembly

Supplementary Movie 3: Adaptability to swarm scale changes

Supplementary Movie 4: Cooperative cargo transportation

Supplementary Movie 5: Environment exploration - Entering a passenger elevator

Supplementary Movie 6: Environment exploration - Exploring a complex maze
